# Patterns of Genetic Variation of *Nepeta nuda* L. from the Central Balkans: Understanding Drivers of Chemical Diversity

**DOI:** 10.3390/plants13111483

**Published:** 2024-05-27

**Authors:** Luka Petrović, Marijana Skorić, Branislav Šiler, Tijana Banjanac, Uroš Gašić, Dragana Matekalo, Tamara Lukić, Jasmina Nestorović Živković, Slavica Dmitrović, Neda Aničić, Milica Milutinović, Jelena Božunović, Biljana Filipović, Miloš Todorović, Danijela Mišić

**Affiliations:** Department of Plant Physiology, Institute for Biological Research “Siniša Stanković”—National Institute of the Republic of Serbia, University of Belgrade, Bulevar despota Stefana 142, 11108 Belgrade, Serbia; luka.petrovic@ibiss.bg.ac.rs (L.P.); tbanjanac@ibiss.bg.ac.rs (T.B.); uros.gasic@ibiss.bg.ac.rs (U.G.); dragana.bozic@ibiss.bg.ac.rs (D.M.); tamara.lukic@ibiss.bg.ac.rs (T.L.); jasmina.nestorovic@ibiss.bg.ac.rs (J.N.Ž.); slavica.dmitrovic@ibiss.bg.ac.rs (S.D.); neda.anicic@ibiss.bg.ac.rs (N.A.); milica.milutinovic@ibiss.bg.ac.rs (M.M.); jelena.boljevic@ibiss.bg.ac.rs (J.B.); biljana.nikolic@ibiss.bg.ac.rs (B.F.); milos.todorovic@ibiss.bg.ac.rs (M.T.)

**Keywords:** *Nepeta nuda*, *Lamiaceae*, genetic variability, chemodiversity, populations, microsatellites, GC-MS, UHPLC/QToF MS

## Abstract

*Nepeta nuda* L., a notable medicinal species in the tradition of the Balkan region, is a rich source of bioactive iridoids and phenolics previously described as high-resolution taxonomical classifiers for the genus *Nepeta*. However, their potential in investigating intra-species differentiation is here described for the first time. The aim was to recognize the sources of natural chemical diversity and their association with the genetic variability both within and among *N. nuda* populations in the Central Balkans. Chemical diversity was assessed from methanol extracts and essential oils through untargeted and targeted metabolomics using state-of-the-art analytical tools, covering a broad spectrum of compounds that represent the *N. nuda* metabolome. We found that chemodiversity primarily resides within populations of *N. nuda*, and similar results were obtained at the DNA level using microsatellite markers. The low genetic and chemical differentiation of the studied *N. nuda* populations implies that their metabolomic profiles may be less influenced by geographic distance and variable environmental conditions within the Central Balkans, as they are under the pivotal control of their genetic backgrounds. Screening the distribution of the major bioactive compounds belonging to phenolics (phenolic acids and flavonoids) and iridoids (both aglycones and glycosylated forms), within and among *N. nuda* populations, is able to guarantee mass spectrometry-based tools for the selection of elite representative genotypes with practical importance. The knowledge acquired will allow us to delve deeper into the molecular background of *N. nuda* chemical diversity, which is the course of our further work.

## 1. Introduction

*Nepeta nuda* L. (fam. *Lamiaceae*) is a widely distributed Eurasian species, traditionally used in folk medicine of the Balkan nations [1], along with other congeneric taxa. This remarkable plant can be found in forest clearings and meadows, at montane and subalpine altitudes up to 2100 m [2], and often in ruderal habitats alongside roads where its robust habitus, represented by colorful long-lasting flower spikes, dominates other species in the surrounding (personal observation). *N. nuda* is traditionally used as a remedy for various ailments such as gastrointestinal problems and respiratory diseases, as well as for treating wounds [3]. Numerous studies have shown that extracts of *N. nuda* have antiviral [4], antioxidant [5], and antibacterial [6,7] properties. In addition, essential oils of *N. nuda* have shown phytotoxic and herbicidal effects [4,8], which make this species potentially useful as a novel source of potent biopesticides. The various biological activities of its extracts and essential oils could be attributed to the two dominant classes of specialized metabolites: terpenoids (mainly monoterpenes and sesquiterpenes) and phenolics (primarily flavonoids and phenolic acids). The most abundant monoterpenes among *Nepeta* species occur either as aglycones or glycosides of iridoids. A characteristic representative of the group of aglycones is nepetalactone, a bicyclic monoterpenoid exclusively found in *Nepeta* species that occurs in the form of different diastereoisomers but also as hydrogenated derivatives such as 5,9-dehydronepetalactone or nepetalic acid. Iridoid glycosides, featured by the presence of a sugar component attached to an iridoid scaffold, are 1,5,9-*epi*-deoxyloganic acid, nepetaside, and nepetariaside (literature survey is summarized in Supplementary Table S1). Chemical composition of *N. nuda* can be influenced not only by the geographical origin and harvesting time but also by environmental parameters such as temperature, precipitation, and solar radiation [3,9,10,11]. Moreover, variable qualitative and quantitative composition of nepetalactone stereoisomers can be found in *N. nuda* essential oils (EOs) of different origins (Supplementary Table S1), which indicates that the metabolome of this species might be highly susceptible to the influence of both endogenous and environmental factors. According to Aćimović et al. [3], nepetalactones represent minor components of the essential oils of *N. nuda* collected in Serbia, while in populations from Greece, Turkey, and Iran, various nepetalactone stereoisomers constitute a large proportion of their essential oils [4,12,13]. The most characteristic phenolic constituents of *N. nuda* are rosmarinic, caffeic, chlorogenic, and ferulic acids, as well as aesculin [10,14,15]. However, chemical profiling of non-volatile compounds such as phenolics or iridoid glycosides has not been extensively performed, as most analyses were focused on the characterization of *N. nuda* essential oils composition (Supplementary Table S1).

*N. nuda* has a wide geographical range [16] and inhabits regions with different climate conditions, soil types, and altitudes, while various biotic and abiotic factors affect its fitness and biomass productivity. Increasing evidence shows that maintaining genetic diversity within natural populations of various plant species can maximize their potential to withstand and adapt to environmental fluctuations [17], although it is difficult to predict the novel selection pressures to which populations will be exposed. The evaluation of the genetic diversity of natural populations of *N. nuda* is directed into providing the evidence on their differentiation magnitude and predicting the likelihood of their survival within the contemporary environmental pressures such as global climate change. Addressing a great challenge of mankind to increase agricultural productivity while preserving and increasing biodiversity [18], we bring into focus the relationships between the genetic and chemical diversity patterns of *N. nuda* populations from the Central Balkans, thus setting the stage for the identification and selection of lines featuring key traits for maximizing the biomass and secondary metabolite production of this medicinal plant. Understanding the reciprocal relationships between the genotype and phenotypic traits in this species will allow us to extend our findings to other congeneric species. Recognition of highly and less-productive populations and genotypes of *N. nuda* will facilitate our quest to explain the molecular background of the chemical diversity of iridoids within the genus *Nepeta*, which is the course of our future work. To the best of our knowledge, no studies dealing with *N. nuda* intra-species chemodiversity with reference to its genetic background in wild populations have previously been conducted, and our intention was to fill these knowledge gaps by adopting the combination of EST-SSR and SSR molecular markers in parallel with state-of-the-art metabolomics tools which are able to encompass different components of the *N. nuda* metabolome. Codominant markers have not been previously employed to estimate the genetic variations of any *Nepeta* sp. at the population level through the estimation of heterozygosity, allelic fixation index, variance fractioning, or population structuring.

## 2. Results and Discussion

Qualitative and quantitative profiling of specialized metabolites, while capturing genotype-dependent variations, enabled us to comprehensively acquire, analyze, and adequately interpret the overall chemical diversity of *N. nuda* in the Central Balkans. State-of-the-art analytical techniques (LC-MS and GC-MS) that cover different parts of the metabolomes of the targeted taxon are well-certified tools to comprehensively assess the underlying chemical diversity and complexity and to aid in predicting differences at inter- and intra-population level [19]. As chemodiversity has its foundations in genetic diversity, we utilized a set of EST-SSR and SSR markers to produce informative DNA data for the reconstruction of the genetic background of *N. nuda* populations which, to our knowledge, has not been undertaken before.

### 2.1. UHPLC-ESI-QToF-MS Identification of N. nuda Leaf Metabolites

**Figure 1:**
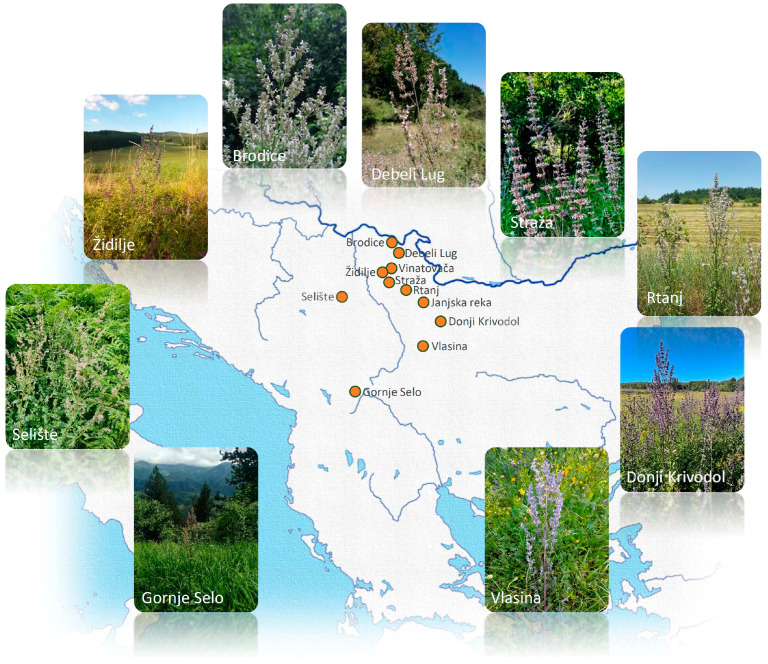
Map representing the populations of *Nepeta nuda* L. originating from the central Balkan Peninsula analyzed in the present study. For the coordinates, please refer to Table 4. Plant photos denote *N. nuda* individuals captured on site.

The subject of this research was the detailed non-targeted characterization of metabolites accumulated in the leaves of *N. nuda*, inhabiting eleven locations in the Central Balkans (Figure 1). The focus was on phenolic and iridoid compounds, which are the two most significant groups of bioactive metabolites in the *Nepeta* species [19,20]. Data on previous records about iridoids and phenolics previously reported for *N. nuda* are summarized in Supplementary Table S1. Using an UHPLC-ESI-QToF-MS instrument operating in both positive and negative modes, seventy-eight compounds were identified, which belong to hydroxybenzoic (ten compounds) and hydroxycinnamic (nineteen compounds) acid derivatives, iridoid glycosides (nine compounds), iridoid aglycones (sixteen compounds), flavonoid glycosides (two compounds), and flavonoid aglycones (six compounds), as well as sixteen compounds from other groups of metabolites.

UHPLC-ESI-QToF-MS data on metabolites identified in the leaf extracts of eleven *N. nuda* accessions are summarized in Table 1. The base peak chromatograms, in negative and positive ionization modes, of 11 different *Nepeta nuda* leaf extracts are depicted in Supplementary Figure S1. References related to the previous identification of these compounds in different *Nepeta* species are listed as well. By searching the available literature, we found that seven compounds (Supplementary Figures S2–S5) listed in Table 1 were identified for the first time in any *Nepeta* species. Supplementary Table S2 summarizes the peak areas of the identified compounds in the eleven investigated extracts.

From the group of ten hydroxybenzoic acid derivatives, four compounds were identified as free acids (**2**, **3**, **6**, and **9**), while the other six, especially abundant, were found to be hexosyl derivatives, giving a specific fragment ion resulting from the loss of 162 Da (hexosyl residue). The fragmentation pathway of compound **5** (vanillic acid hexoside), for which we found no data, confirming that it has not previously been identified in any *Nepeta* species, is shown in Supplementary Figure S2A.

All nineteen compounds from the group of hydroxycinnamic acids, except for coumarin umbelliferone (compound **16**), are actually different derivatives of caffeic acid. The largest number of these derivatives were identified as esters with tartaric, tartronic, glycolic, or malic acids, which are otherwise common for *Nepeta* species [24,25,26,29]. Compound **25**, which was not previously identified in *Nepeta* species, with the molecular ion at 309 *m*/*z*, showed specific MS^2^ ions corresponding to deprotonated ferulic acid (193 *m*/*z*) and its fragments (178, 147, 134, and 117 *m*/*z*) (Supplementary Figure S2B).

**Figure 2:**
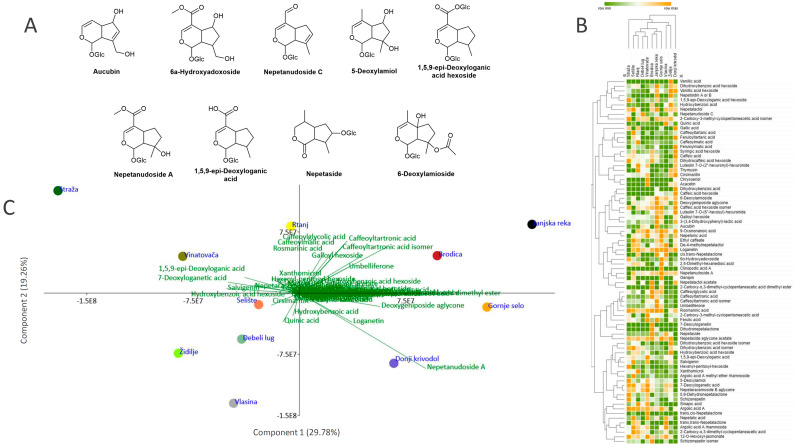
(**A**) Chemical structures of characteristic iridoid glycosides identified in *Nepeta nuda* methanol extracts (Glc—glucose or another hexose). (**B**) Heatmap of the scaled QToF MS data with the samples arranged according to the hierarchical cluster analysis (Spearman’s method of cluster agglomeration). (**C**) Principal component analysis (PCA) biplot constructed based on the QToF MS data, with the first two PCs explaining 49.04% of the total variance among *N. nuda* accessions. Participation of the variables (compounds) in the first two PCs is indicated by the corresponding loading plots.

Nine derivatives of iridoid glycosides were identified in negative ionization mode (Figure 2A). Some compounds showed [M–H]^−^ as a molecular ion, while in three iridoid glycosides (**32**, **34**, and **35**), an adduct with formic acid ([M +HCOOH–H]^−^) was observed, giving a shift in the molecular ion mass of 46 Da. The most abundant compound, compared by peak area, was nepetanudoside A (compound **35**). This compound, typical for *N. nuda* [33], was detected in all but one population (Straža) in our research. One of the derivatives of iridoid glycoside (compound **33**, 5-deoxylamiol) has not been previously reported in any *Nepeta* species but is typical for *Lamium* species from the same family [52]. The proposed detailed fragmentation pathway of this compound is shown in Supplementary Figure S3.

Iridoid aglycones were mostly detected in the positive ionization mode due to their polarity, except for 7-deoxyloganetin and nepetalic acid (compounds **49** and **50**), which gave more abundant peaks in the negative ionization mode. Compound **49** was identified in only one population (Brodica), while compound **50** was detected in all populations. Out of a total of sixteen identified compounds, the presence of fourteen was confirmed by comparison with the available literature on *Nepeta* species (Table 1), while genipin and loganetin (compounds **39** and **42**) were identified through the evaluation of their MS data. Supplementary Figure S4A represents the detailed fragmentation pattern in the positive ionization mode of genipin (compound **39**), which was detected in three populations: Selište, Donji Krivodol, and Gornje Selo. Compound **39** showed MS^2^ the base peak at 103 *m/z* in the positive ionization mode, which was in accordance with literature data [53]. The iridoid loganetin (compound **42**), which was not previously reported as common for the genus *Nepeta*, was detected in almost all populations (except for Straža) in considerable amounts. Its chemical structure and explanation of the formation of all MS^2^ fragments is shown in Supplementary Figure S4B.

The vast majority of *Nepeta* species studied by Jamzad et al. [43] contains flavone-type flavonoids on the leaf surface. In the case of flavonoid glycosides, only two luteolin derivatives (compounds **55** and **56**) were identified in our study, and both were previously reported in catnip [24,27]. Our results also show the presence of six flavonoid aglycones belonging to this subgroup of flavonoids, while derivatives from other subgroups were not identified. By comparison of the peak areas of these compounds, it can be noticed that salvigenin (compound **62**) was very abundant flavone. This compound was previously reported to be present only in the aerial parts of *N. transcaucasica* [44], in *N. nuda*, *N. assurgens*, *N. asterotricha*, *N. crispa*, *N. pungens*, *N. saturejoides* [43], and *N. baytopii* [29].

Ungrouped metabolites do not belong to any of the aforementioned classes. Two fatty acids (compounds **69** and **73**) and four carboxylic acids (compounds **70**, **72**, **74**, and **78**) specific to the genus *Nepeta* were recorded (Table 1). Compound **75**, with the molecular ion at 201 *m*/*z*, was identified as argolic acid A. It gave the MS^2^ base peak at 139 *m*/*z*, resulting from the loss of H_2_O and CO_2_ (62 Da). This cyclopentane–monoterpene diol was named after the species from which it was first isolated, *N. argolica* subsp. *argolica* [51]. In our study, two new derivatives of argolic acid A were found and marked as argolic acid A rhamnoside and argolic acid A methyl ether rhamnoside (compounds **71** and **76**, respectively). Their structures and fragmentation pathways are proposed in Supplementary Figure S5.

To visualize relations in metabolic fingerprints of eleven *N. nuda* populations, HCA was performed based on the Spearman’s method of cluster agglomeration. Population Donji Krivodol is clustered separately from other populations on the HCA plot (Figure 2B), owing to the accumulation of high amounts of caffeic acid and its derivatives. The remaining populations are separated into two major subclusters, the first one containing only the Straža population, characterized by high amounts of nepetalactone-like compounds. Populations belonging to the second subcluster are separated into two groups, the first one containing the populations Vlasina and Židilje and the second containing the remaining populations. PCA analysis segregated the populations Janjska reka, Gornje Selo, Brodica, and Donji Krivodol from the remaining ones along the PC1, which explains 29.78% of the total variability (Figure 2C). The major contributors to the diversification along PC1 are nepetanudoside A, caffeoyltartonic acid, 7-deoxyloganetic acid, salvigenin, and 1,5,9-*epi*-deoxyloganic acid. Nepetanudoside A, caffeoyltartronic acid, and caffeoylglycolic acid are the major factors behind the diversification along PC2, which explains 19.26% of the total variability and mostly affects the population Vlasina. PC1 and PC2 cumulatively explain 49.04% of the variability.

### 2.2. GC-MS Non-Targeted Metabolomics of N. nuda EOs

In total, 30 terpene compounds were identified in the EOs of *N. nuda*, adopting the GC-MS analysis (Table 2).

**Figure 3:**
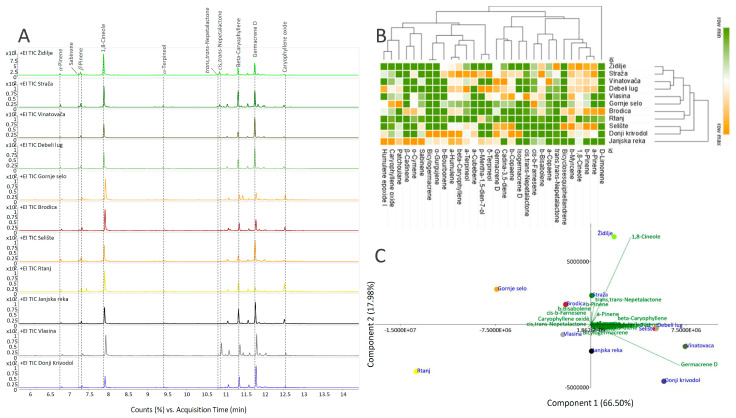
(**A**) Representative TIC GC/MS chromatograms of 11 samples of EOs originating from Central Balkans populations of *Nepeta nuda*. (**B**) Heatmap of the scaled GC/MS data with the samples (both populations and compounds) arranged according to the hierarchical cluster analysis and adopting the Spearman’s method of cluster agglomeration. (**C**) Principal component analysis (PCA) biplot constructed based on the GC/MS data, with the first two PCs explaining 84.48% of the total variance among *N. nuda* accessions. Participation of the variables (compounds) in the first two PCs is indicated by the corresponding loading plots.

These compounds belong to the groups of monoterpenes (6 monoterpenes and 6 oxygenated monoterpenoids) and sesquiterpenes (16 sesquiterpenes and 2 oxygenated sesquiterpenoids). The representative GC-MS TIC chromatograms are shown in Figure 3A. Among monoterpenoids, 1,8-cineole (**7**), *α*-pinene (**1**), and *β*-pinene (**3**) predominated. From the group of monoterpenoid iridoids, two nepetalactone stereoisomers were detected: *trans*,*trans*- (**11**) and *cis*,*trans*-nepetalactone (**12**), albeit in low amounts. Interestingly, 1,5,9 -dehydronepetalactone and dihydronepetalactones, as well as their derivatives, were not recorded in the EOs of *N. nuda* through the GC-MS analysis. The same applies for the groups of diterpenes and triterpenes. This can be ascribed, at least partially, to the hydrodistillation method for the EO isolation but also to the HS sampling procedure, which includes the incubation of samples at 40 °C prior to sampling and injection. This temperature might be too low to induce the evaporation of these compounds, which might generally result in a lower abundance of less volatile compounds. beta-Caryophyllene (**17**) and Germacrene D (**23**) were detected as the major sesquiterpenoids in the majority of analyzed *N. nuda* EOs, while *α*-humulene (**22**) and *β*-bisabolene (**24**) were also present in significant amounts.

All the analyzed EOs of eleven *N. nuda* populations displayed a similar chemotype characterized by the predominance of 1,8-cineole, germacrene D, and beta-caryophyllene. As the major constituents of *N. nuda* EOs, other studies reported caryophyllene oxide [54], camphor and 1,8-cineole [55], caryophyllene [56], nepetalactone [4,57,58], 1,8-cineole and 4a*α*,7β,7a*α*-nepetalactone [59], nepetalactone and germacrene [6], 1,8-cineole [60], and 1,8-cineole and a mixture of nepetalactones and germacrene-D [61]. Previously reported data on the composition of terpenes in *N. nuda* EOs are shown in Supplementary Table S1. These differences might arise from many factors, including plant origin and growth conditions, growth stage and plant parts analyzed, the subspecies or a cultivar in question, EO isolation procedure, and GC-MS conditions. When the composition of EOs of *N. nuda* sampled at Mt Rtanj (Serbia) was tracked through three successive years, the content and ratio of EO components were found to be slightly influenced by weather conditions such as temperature, precipitation, and insolation [62]. The same authors proposed four chemotypes of *N. nuda* EOs: (1) nepetalactone; (2) 1,8-cineole; (3) mixed (nepetalactone+1,8-cineole+germacrene D); and (4) nonspecific chemotypes. Based on this principle, the Rtanj population within the present study, rich in 1,8-cineole, belongs to the chemotype 2. A previous study [11] revealed 4a*α*,7*β*,7a*α*-nepetalactone, 1,8-cineole, and germacrene D as the most abundant volatile compounds in *N. nuda* plants grown under either in vitro or ex vitro conditions. Caryophyllene, *β*-ocimene, bicyclogermacrene, *β*-pinene, myrcene, and humulene were also found to be abundant. Although a previous study reports the comparative analysis of EO composition in four *N. nuda* populations from Iran [13], the present study represents the first successful characterization of interpopulation variability in the qualitative EO composition of this species.

The relative GC-MS quantitative data (peak areas) of eleven *N*. *nuda* EOs were subjected to both PCA and HCA to visualize the relations among and between populations from the Central Balkans (Figure 3B and Figure 3C, respectively). The *N. nuda* EO sample originating from Janjska reka clustered separately from the remaining populations on the HCA plot constructed based on the Spearman algorithm, and this was mostly owing to its high accumulation of sabinene, *β*-cadinene, caryophyllene oxide, humulene epoxide I, and *o*-cymene (Figure 3B). The remaining populations grouped into two clusters, the first one containing only the populations Donji Krivodol and Selište, abundant in beta-caryophyllene, *α*-humulene, and *β*-copaene, while the second cluster diversified into two subclusters: one containing the populations Rtanj and Brodica, and the other comprising populations rich in 1,8-cineole, *trans,trans*-nepetalactone, and *α*-copaene, (Vlasina, Gornje Selo, Brodica, Židilje, Straža, Debeli Lug, Vinatovača, and Selište). As revealed by the PCA, the first two PCs cumulatively explained 84.48% of the total variability, with PC1 contributing 66.50% and PC2 17.98%. Populations Vinatovača, Donji Krivodol, Debeli Lug, Selište, and Židilje were clearly separated from Rtanj, Gornje Selo, Brodica, and Vlasina along the PC1 (Figure 3C). The major contributors to the diversification of populations along PC1 were germacrene D, 1,8-cineole, and beta-caryophyllene, and along PC2, it was 1,8-cineole and germacrene D (Figure 3C).

### 2.3. Targeted qqqMS Profiling of Major Iridoids and Phenolics in N. nuda Methanol Extracts

To estimate inter- and intra-population phenotypic diversity in the quantitative composition of major phenolics and iridoids in *N. nuda*, and thus the productivity of individual genotypes, we further applied a targeted metabolomics approach. Selected phenolics (six compounds) and iridoids (three compounds) were previously adopted as high-resolution taxonomic classifiers to discriminate *Nepeta* species [20]. The set selected for the present study comprised a total of 12 compounds from the group of iridoids (four compounds) and phenolics (eight compounds). Although some previous studies revealed that the metabolic profiles of phenolics and iridoid glycosides depend on variable growth conditions [10], this is the first report on intra- and inter-population comparative analysis of these metabolites in *N. nuda*.

**Figure 4:**
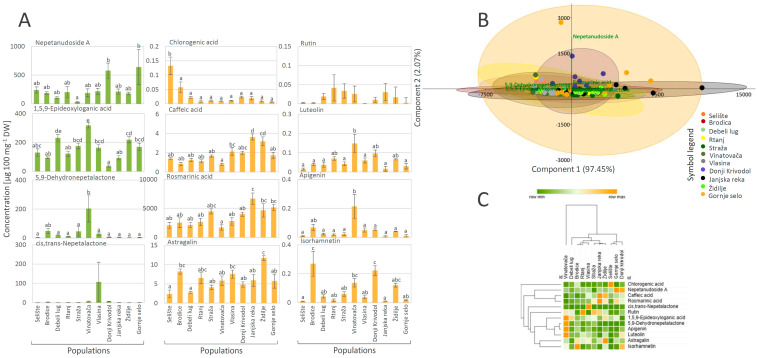
(**A**) UHPLC/(±)HESI-MS^2^ quantitative data of targeted iridoids (green bars) and phenolics (orange bars) in methanol extracts of *Nepeta nuda* originating from 11 Central Balkans populations. Mean values ± SE are presented. Values labeled with different letters are significantly different (*p* < 0.05) according to post hoc Tukey’s test of one way ANOVA. (**B**) Principal component analysis (PCA) biplot constructed based on the quantitative data, with the first two PCs explaining 99.52% of the total variance among *N. nuda* accessions. For eleven *N. nuda* populations, 95% confidence ellipses are presented, colored according to the symbol legend. Participation of the variables (compounds) in the first two PCs is indicated by the corresponding loading plots. (**C**) Heatmap of the scaled UHPLC/(±)HESI-MS^2^ quantitative data with populations and compounds arranged according to the hierarchical cluster analysis and adopting the Pearson’s method of cluster agglomeration.

The UHPLC/DAD/(±)HESI-MS^2^ analysis was targeted towards four iridoids, two of them in the form of aglycones (*cis*,*trans*-nepetalactone and 5,9-dehydronepetalactone) and two in the glycosylated form (1,5,9-*epi*-deoxyloganic acid and nepetanudoside A), as well as towards seven phenolic compounds belonging to the groups of phenolic acids (rosmarinic, caffeic, and chlorogenic acid) and flavonoids (luteolin, apigenin, rutin, and astragalin). These compounds were selected based on previous studies reporting their high abundance in *N. nuda* [10,14,15,20]. Validation parameters of the adopted UHPLC/DAD/(±)HESI-MS^2^ method are presented in Supplementary Table S3. Among the analyzed iridoids, nepetanudoside A and 1,5,9-*epi*-deoxyloganic acid were the most abundant compounds in the majority of the assessed *N. nuda* populations (Figure 4A).

Nepetanudoside A amounts were the highest in the populations Donji Krivodol (~578 µg 100 mg^−1^ DW) and Gornje Selo (~641 µg 100 mg^−1^ DW), while the lowest amounts were recorded in methanol extracts of *N. nuda* population originating from the locality Straža (~29 µg 100 mg^−1^ DW). The highest amounts of 1,5,9-*epi*-deoxyloganic acid were recorded in the *N. nuda* population Vinatovača (~318 µg 100 mg^−1^ DW), and the lowest in Donji Krivodol (~36 µg 100 mg^−1^ DW). Methanol extracts of plants from Vinatovača were especially rich in 5,9-dehydronepetalactone (~204 µg 100 mg^−1^ DW), while the population Vlasina displayed high amounts of *cis*,*trans*-nepetalactone (~108 µg 100 mg^−1^ DW). In the majority of analyzed populations, *cis*,*trans*-nepetalactone was recorded in only a few genotypes, usually in low amounts.

Among the targeted phenolic compounds, rosmarinic acid was the most abundant in all analyzed populations (Figure 4A). This was in accordance with previous studies, which highlighted rosmarinic acid as the major phenolic compound in *N. nuda* [10,14,15,20,63] and other *Nepeta* species [19,20,64,65]. Samples from Janjska reka displayed the highest amounts of this phenolic acid (6698 µg 100 mg^−1^ DW) followed by samples from Gornje Selo, Židilje, and Straža (Figure 4A). A similar trend was observed for caffeic acid, where Janjska reka displayed the highest amounts of this compound (~4 µg 100 mg^−1^ DW). Chlorogenic acid was the most abundant in samples from Selište, with average values reaching 0.13 µg 100 mg^−1^ DW. Among the analyzed flavonoids, astragalin was the most abundant, especially in samples from the population Židilje (~11.70 µg 100 mg^−1^ DW). Samples originating from Vinatovača were the richest in apigenin (0.21 µg 100 mg^−1^ DW) and luteolin (0.15 µg 100 mg^−1^ DW). As for rutin, the amount of this compound showed prominent variability within populations; thus, no significant differences in the average amounts were recorded between populations.

To evaluate the variability in the quantitative composition of iridoids and phenolics in *N. nuda* within the Central Balkans area, PCA was performed, adopting Ward’s method of the data agglomeration (Figure 4B). The major contributors to the diversification of samples along the PC1, explaining 97.45% of the total variability, are *cis*,*trans*-nepetalactone, rosmarinic acid, 5,9-dehydronepetalactone, and 1,5,9-*epi*-deoxyloganic acid. As for the PC2, which contributes to the total variability with 2.07%, nepetanudoside A is the major contributor to the diversification along this component. According to the HCA plot (Figure 4C), constructed via Pearson’s algorithm, population Vinatovača was segregated from the other populations, as it was the richest in 1,5,9-*epi*-deoxyloganic acid, 5,9-dehydronepetalactone, apigenin, and luteolin. The remaining populations were grouped into two subclusters, the first one contained populations Donji Krivodol, Gornje Selo, and Selište, while the second contained the remaining seven populations. Interestingly, the results display high intra-population variability, especially for Janjska reka, Gornje Selo, and Brodica.

Although iridoids are probably the most potent bioactive compounds in *Nepeta* species, studies dealing with simultaneous analysis of iridoid aglycones and their glycosides are scarce, most probably due to different extractions procedures and analytical techniques required for their precise identification and quantification. Here, we adopted cutting-edge analytical instrumentation to analyze in parallel iridoid aglycones and glycosides in *N. nuda* accessions. When compared to other congeneric species, *N. nuda* can be described as a low producer of iridoid aglycones (nepetalactones and their derivatives) but a relatively significant producer of glycosylated iridoids. Interestingly, genotypes containing iridoid aglycones in trace amounts are also present among the analyzed populations, which might have important consequences on their performance in natural habitats in the way they chemically respond to biotic constraints. The observed diversity in iridoid content might be due to the genotype-dependent regulation of iridoid biosynthesis in *N. nuda*, which, in other *Nepeta* species, usually occurs at the transcriptional level and affects the expression of biosynthetic genes and the corresponding transcription factors [66,67]. Our further work is aimed at deciphering the regulatory mechanisms that define the metabolic flux through the iridoid pathway branches leading to nepetalactone and its derivatives or to iridoid glycosides, among which nepetanudoside A and 1,5,9-*epi*-deoxyloganic acids predominate. This will be assisted by simultaneous metabolic profiling and gene co-expression analysis of the highly and less-productive *N. nuda* genotypes identified within the present study. The targeted metabolic profiling of major iridoids and phenolics indicated that some of individuals/genotypes were characterized by unique metabolic profiles, which may be selected for further profiling of expression of the genes related to the biosynthesis of iridoids and rosmarinic acid. This is the course of our further work.

On the other hand, *N. nuda* is a rich source of rosmarinic acid, previously reported to be the most abundant phenolic compound among *Nepeta* species. Within the present study, it was possible to identify *N. nuda* genotypes rich in rosmarinic acid, which will be used in our quest for the factors regulating the metabolism of this phenolic acid. Regulatory mechanisms most likely vary across different biosynthetic pathways, such as terpenoids and phenolics (Zhiponova, M. personal communication), and exploring the correlations between these two classes of metabolites, at the level of metabolomes, transcriptomes, and proteomes, may support the development of computational models that aim to link chemodiversity to genetic and evolutionary principles.

### 2.4. Parameters of Genetic Variation within and among the Populations

It has been well documented that genetic background plays a key role in defining the composition and overall yield of specialized metabolites in plants. However, genotype and environment interactions determine the overall composition of metabolites at multiple levels of biological organization. The aim of the current study was to develop a knowledge resource for *N. nuda* chemodiversity, with reference to the genetic background in wild populationstowards facilitating future utilization for commercial and pharmacological applications.

To elucidate the fundamentals of species’ natural chemical diversity, we assessed the genetic structure of wild populations, adopting a set of EST-SSR molecular markers mined from a leaf transcriptome of *N. nuda* and a pair of previously published SSR markers. Microsatellites, being highly polymorphic codominant markers with a neutral evolutionary history, provide a basic knowledge of the species’ genome variation through the estimation of the allelic fixation index, as well as the structure of populations, offering outstanding benefits because of their low cost and user-friendly nature [68]. EST-SSRs markers, being transcriptome-derived, are considered less variable as compared to genomic microsatellites, but meticulous selection of loci to be used can result in a greater discriminating power in comparison to genomic SSRs [69]. To the best of our knowledge, no prior attempts to characterize genetic diversity of any taxon belonging to the genus *Nepeta* employing microsatellites was made. Previously, ISSR and RAPD molecular markers were utilized to investigate intraspecific and intrageneric genetic variations in several *Nepeta* taxa [70,71,72,73,74]. DNA barcoding regions, including nuclear (ITS) and plastid (e.g., *mat*K, *rbc*L, *trn*L-F, *trn*S-*trn*G, and *psb*J-*pet*A) markers, were often utilized to reconstruct phylogenetic relations within the genus *Nepeta* [75,76,77]. Petrova et al. [10] performed DNA barcoding by analyzing the *N. nuda* ITS sequences and regions of plastid DNA (*rbc*L/*mat*K/*trn*H). Comparison of the obtained ITS sequences of *N. nuda* with the corresponding sequences of other *Nepeta* species revealed that it closely clustered with *N. sheilae, N. deflersiana, N. isaurica*, *N. congesta*, *N. heliotropifolia*, *N. schiraziana*, and *N. cataria*. However, three chloroplast markers showed its close relation to *N. italica*, *N. tuberosa*, *N. cataria*, *N. grandiflora*, and *N. hemsleyana*. Although both ITS and plastid markers have been proved to be suitable for determining taxonomic relationships among *Nepeta* species, they are considered too conservative to represent genetic variability within species and have thus not been considered for the present study.

The total number of alleles across the nine microsatellite markers used within the present study ranged from 2 for Cont039-gene0.9 to 12 for MN26. The basic parameters of genetic variation within and among the studied *N. nuda* populations are presented in Table 3.

The mean number of alleles in a population ranged from 2.222 in the population Selište to 3.444 in the population Židilje, which is slightly higher than in *Origanum compactum* L. from Morocco [78] but considerably lower than in *Rosmarinus officinalis* L. from eastern Iberian Peninsula [79] and *Salvia officinalis* L. from the Balkans [80]. The number of private alleles was low and ranged from 0 to 3, the latter being scored in the population Vlasina, which is in the range of the results obtained for several species from the same family [78,80]. Observed heterozygosity ranged from 0.395 in Debeli Lug to 0.544 in Židilje, higher than that reported for *O. compactum* [57] but considerably lower than for *R. officinalis* [79] and *S. officinalis* [80]. This parameter revealed a high heterozygosity level within individuals. Expected heterozygosity was generally slightly lower, with a span from 0.358 (uHe = 0.386) in Brodica to 0.524 (uHe = 0.589) in Gornje Selo, while Shannon’s information index varied from 0.593 in Brodica to 0.931 in Gornje Selo. All the populations showed heterozygote excess, having negative F values, and only Debeli Lug had a slight heterozygote deficiency (F = 0.05).

**Figure 5:**
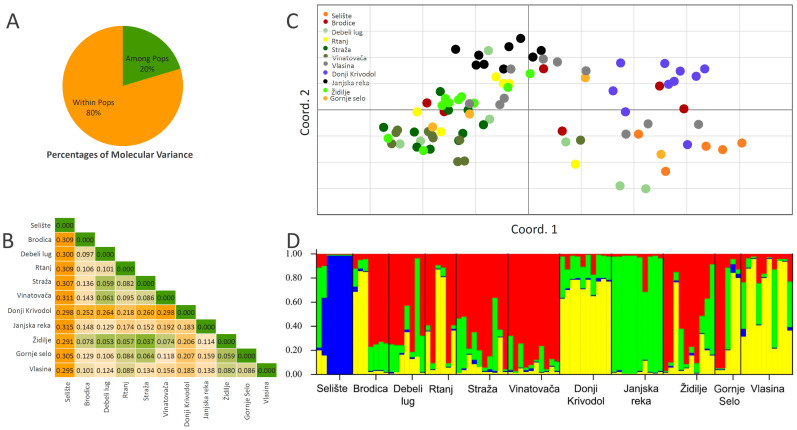
Genetic diversity of *Nepeta nuda* populations from the Central Balkans, as estimated by EST-SSR and SSR markers. (**A**) Analysis of molecular variance. (**B**) Pairwise F_ST_ values of eleven populations of *N. nuda*. The green-to-orange color scale denotes low-to-high genetic differentiation. (**C**) PCoA biplot representing genetic clustering of individuals belonging to eleven populations of *N. nuda*. Cumulative variation explained by the two coordinates is 29.33% (19.34% COORD.1 + 9.99% COORD.2). (**D**) Individuals’ recruitment of the four genetic clusters inferred by STRUCTURE.

The AMOVA across all loci revealed that the greatest amount of genetic variance is captured within populations (80%), and among-population variance contributed 20% to the total variation (Figure 5A). This is quite an ordinary rule for outcrossing species belonging to the *Lamiaceae* family, as 78% and 84.73% within-population variation was recorded for *O. compactum* [78] and *S. officinalis* [80], respectively. Weir and Cockerham’s [81] estimation of F_ST_ was 0.203, which was significantly different from zero and similar to the value generated by AMOVA.

Pairwise F_ST_ values between populations are presented in Figure 5B in colors ranging from green (no genetic differentiation) to orange (high genetic differentiation). Each value with F_ST_ > 0.15 may imply significant population differentiation [64]. It can be seen that the populations Selište and Donji Krivodol are rather strongly differentiated from the remaining populations, although they were not significantly different based on the chemical composition.

In Figure 5C, a two-dimensional PCoA scatterplot displays a grouping of individuals according to the population affiliation. It can be noted that the populations Vlasina, Gornje Selo, Brodica, and Debeli Lug failed to group along the two coordinates (Figure 5C).

The Mantel test revealed weak and a non-significant correlation (r = 0.3019, *p* ≤ 0.119) between geographic and genetic distances.

STRUCTURE Harvester illustrated that K = 4, K = 6, and K = 7 are the most likely scenarios. In Figure 5D, the scenario with four genetic pools is presented. It can be seen that five out of six individuals from the population Selište draw mostly from a separate genetic cluster (blue), and that all individuals from Janjska reka almost completely consist of only one genetic cluster (green). The dominant genetic cluster in most of the remaining individuals is that marked red, while all the individuals from the populations Donji Krivodol and several individuals from Brodica, Rtanj, Židilje, Gornje Selo, and Vlasina draw mostly from the genetic pool marked yellow.

The analyzed set of EST-SSR and SSR markers proved not only to be very useful for the genetic diversity studies at the intra-species level but could possibly be transferred to other taxa of the genus *Nepeta*. The developed markers can further assist the germplasm conservation of *N. nuda* across its area.

## 3. Materials and Methods

### 3.1. Collection and Preparation of Plant Material

Aerial parts of flowering *N. nuda* plants were collected in June-July 2022 from 11 populations across Serbia (Figure 1). Plants were identified in the field by the authors. One plant from each population (except for the populations Rtanj, Židilje, and Gornje Selo) was stored in the Herbarium of the Institute of Botany and Botanical Garden, University of Belgrade, BEOU (acronym follows [82] and voucher numbers are presented in Table 4, along with the corresponding locations’ names and their geographic coordinates). For DNA extraction and the preparation of methanol, extracts 2–3 leaves from at least 5 (mostly 10) plants per population were separately disposed into plastic zip-bags containing silica gel. Thus, each population was represented by 5 to 10 biological replicates (individuals/genotypes). Samples were kept in the dark at room temperature until use. For the isolation of essential oils, aerial parts (leaves, stems, and flowers) belonging to each population were pooled, air-dried at room temperature until constant weight was achieved, and subsequently mechanically chopped.

### 3.2. Identification and Quantification of Metabolites in Methanol Extracts of N. nuda

#### 3.2.1. Chemicals and Reagents

Acetonitrile (Fisher Scientific, Leicestershire, UK) and formic acid (Merck, Darmstadt, Germany) were of MS grade. Ultra-pure deionized water was generated using the Water Purification System (New Human Power I Integrate, Human Corporation, Republic of Korea). Standards of *cis*,*trans*-nepetalactone, *trans*,*cis*-nepetalactone, 1,5,9-*epi*-deoxyloganic acid, and 5,9-dehydronepetalactone were isolated from natural sources as previously described by [19]. Nepetanudoside A was quantified based on the calibration curve of loganin. Commercially available analytical standards of loganin, rosmarinic acid, caffeic acid, chlorogenic acid, and rutin (Sigma Aldrich, Hamburg, Germany) were used for quantification purposes.

#### 3.2.2. Preparation of *N. nuda* Methanol Extracts

Silica gel-dried leaves (100 mg) of *N. nuda* were ground in liquid nitrogen and extracted with 1 mL of 96% methanol (*w*:*v* = 1:10). The extraction was performed by vortexing for 1 min and subsequent ultrasound-assisted extraction for 1 h at 4 °C. Supernatants, separated after centrifugation at 10,000× *g* for 10 min, were filtered through cellulose filters with 0.2 µm pore size (Agilent Technologies, Santa Clara, CA, USA).

#### 3.2.3. Characterization of *N. nuda* Leaf Metabolites Using UHPLC-ESI-QToF-MS Analysis

One randomly selected *N. nuda* sample from each population was subjected to metabolic fingerprinting adopting a high-resolution mass spectrometry approach (HRMS). Analyses were performed using an Agilent 1290 Infinity UHPLC system in combination with a quadrupole time-of-flight mass spectrometer (6530C Q-QToF-MS, Agilent Technologies, Inc., Santa Clara, CA, USA). The mass detector was equipped with an electrospray ionization (ESI) source that was operated in both positive and negative ionization modes in the mass range from 100 to 1000 *m*/*z*. Chromatographic separation was performed at 40 °C on a Zorbax C18 column (2.1 × 50 mm, particle size 1.8 µm; Agilent Technologies, Inc., Santa Clara, CA, USA). The gradient elution program, UHPLC and MS parameters, as well as ion source settings, were described by Kostić et al. [83] and implemented in analyses of *Nepeta* species [66].

Agilent MassHunter software was used for acquisition, control, and MS data collection from the 6530C QToF-MS instrument. For the evaluation and presentation of MS data, R Studio software (*enviPick* and *xcms* R packages) was used as previously reported [84]. Metabolites were identified based on their monoisotopic masses and MS^2^ fragmentation and confirmed using previously reported metabolomic data about *Nepeta* species [19,27,30,32]. Accurate masses of components and fragment ions were calculated using ChemDraw software (version 12.0, CambridgeSoft, Cambridge, MA, USA). The CAS SciFinder-n database was used to search for chemical compounds by formulae and structures (https://scifinder-n.cas.org/ (accessed on 12 January 2024)).

#### 3.2.4. UHPLC/DAD/(±)HESI-MS^2^ Targeted Metabolic Profiling

Targeted UHPLC/DAD/(±)HESI-MS^2^ metabolomics approach was adopted to quantify individual phenolics and iridoids in *N. nuda* methanol extracts. Analyses were performed on a Dionex UltiMate 3000 UHPLC system coupled with a DAD detector and configured with a triple quadrupole mass spectrometer (TSQ Quantum Access Max, Thermo Fisher Scientific, Basel, Switzerland). Samples were chromatographically separated on a Hypersil gold C18 analytical column (50 × 2.1 mm, 1.9 µm particle size; Thermo Fisher Scientific, Waltham, MA, USA) using the mobile phase gradient and a flow rate previously described by [19]. The mobile phase consisted of (A) water + 0.1% formic acid and (B) acetonitrile + 0.1% formic acid. The injection volume was 10 µL. The selected reaction monitoring (SRM) mode of the instrument was used for the quantification of the targeted compounds by direct comparison with the commercial standards. Calibration curves of pure standards revealed good linearity, with r^2^ values exceeding 0.99 (peak areas vs. concentration). Method validation parameters are presented in Supplementary Table S3. The total amount of each phenolic and iridoid compound was evaluated by the calculation of its peak area and is expressed as µg per 100 mg leaf dry weight (DW).

### 3.3. Determination of Metabolites in Essential Oils of N. nuda Populations

#### 3.3.1. Hydrodistillation of Essential Oils (EOs) from *N. nuda* Aboveground Parts

Essential oils were prepared from aboveground flowering plants via hydrodistillation in a Clevenger-type apparatus connected to a 2-l borosilicate glass flat bottom flask (Isolab, Eschau, Germany), as previously described by [85]. Hydrodistillation was performed for 2 h, using 50–100 g plant material and 1.5 l deionized water (Water Purification System, New Human Power I, Human Corporation, Seoul, Republic of Korea). The essential oil extraction procedure yielded from 2.19 to 5.97 μL essential oil per g dry weight (DW), depending on the *N. nuda* population (Table 2).

#### 3.3.2. GC-MS Non-Targeted Metabolomics of *N. nuda* EOs

Profiling of volatile compounds contained in EOs of *N. nuda* plants was performed using an Agilent 8890 gas chromatography (GC) system coupled with a Mass Selective Detector (5977B GC/MSD, Agilent Technologies, Santa Clara, CA, USA) and connected to an automated sample extraction and enrichment platform (Centri^®^, Markes International Ltd., Bridgend, UK). Chromatographic separations were performed for 21 min on an HP-5MS column (30 m × 0.25 mm, 0.25 μm film thickness; Agilent Technologies, Santa Clara, USA), using helium (99.999%, The Linde Group, Ireland) as a carrier gas at a flow rate of 1.6 mL min^−1^. The settings of the MS procedure were previously described by Aničić et al. [66]. In short, the transfer line was heated at 280 °C and the detector temperature was set to 270 °C. Mass spectra were acquired in the positive EI mode (+70 eV), with the temperature of the EI source set to 280 °C. Column temperature was linearly programmed from 40 to 300 °C at a rate of 20 °C min^−1^ and held isothermally at 240 °C for the subsequent 5 min. Centri^®^ was operating in a direct HS mode. Following dilution of EOs in 96% methanol (5 µL ml^−1^), 10 µL of an EO solution was transferred into a headspace (HS) vial and subsequently incubated at 40 °C for 10 min with agitation at 300 rpm. Sampling was performed in a split mode (20:1), with split flow of 24 mL min^−1^, and the injector temperature set to 250 °C. Analyses were performed in the SCAN mode, tracking the compounds within the range of 45 to 500 amu. The constituents of the reaction mixtures were identified by comparison of their mass spectra and retention times with those of the respective standards, as well as by comparison with the NIST05 library.

#### 3.3.3. Statistical Analysis of Metabolomics Data

For hierarchical cluster analysis (HCA), the input variables were scaled from 0 to 1. Principal component analysis (PCA) was constructed using the Past 4 software (version 4.14; [25,86]. HCA was performed based on either Spearman’s or Pearson’s method of cluster agglomeration, using the Morpheus software (https://software.broadinstitute.org/morpheus (accessed 12 January 2024)). Quantitative metabolomics data were subjected to Tukey’s *post hoc* test (*p* < 0.05) of one-way ANOVA.

### 3.4. Genetic Assessment of N. nuda Populations

#### 3.4.1. EST-SSR Mining

For the RNA-Seq analysis of *N. nuda* leaf trichomes, leaf samples were collected in June 2020 from Balta Berilovac (Stara planina, East Serbia). Trichomes were harvested from fresh leaves using the dry ice abrasion technique, as detailed by [87]. Subsequently, total RNA was isolated from detached trichome samples, and libraries were prepared by Macrogen Europe BV (Amsterdam, The Netherlands) using TruSeq Stranded Total RNA LT Sample Prep Kit (Plant) on the Illumina NovaSeq 6000 platform. RNA-Seq data were searched for microsatellite motifs (SSRs) using the Imperfect SSR Finder software [88]. Motifs of three-to-five nucleotides in size and a minimum of five contiguous repeat units were screened. Primer pairs were designed using Primer3Plus with default settings [89].

#### 3.4.2. DNA Extraction and Optimization of EST-SSR Markers’ Amplification

DNA was isolated from silica gel-dried leaf samples using a modified CTAB method [90]. DNA concentration and purity were assessed spectrophotometrically by measuring the absorbance at 230, 260, and 280 nm (NanoPhotometer N60, Implen, München, Germany).

We selected 20 putative EST-SSR markers to test for their variation in *N. nuda*. In total, thirty-three individuals from eleven *N. nuda* populations (three individuals per population) were used as a panel for the validation of the developed EST-SSR markers. Optimization of primers’ annealing temperatures and amplification of EST-SSR markers were carried out using Mastercycler Nexus Gradient thermal cycler (Eppendorf AG, Hamburg, Germany). PCR reactions were performed in a final volume of 10 µL, each reaction containing 30 ng template DNA, 5.2 µL DreamTaq Green PCR Master Mix (2×; Thermo Fisher Scientific, Karlsruhe, Germany), 0.2 µM forward and reverse primer each, BSA 0.6 µg µL^−1^ (albumin, bovine serum; Sigma-Aldrich, Hamburg, Germany), and additional MgCl_2_ to a final concentration of 4.5 Mm (Thermo Fisher Scientific, Karlsruhe, Germany). All EST-SSR loci were amplified using the following PCR reaction program: 94 °C for 10 min; 36 cycles of 94 °C for 1 min; 52.5–61.0 °C (depending on the calculated annealing temperature for primer pair, see Table 2) for 45 s; 72 °C for 45 s; and 72 °C for 10 min as a final extension step.

Twenty PCR-amplified EST-SSR loci of the 33 selected *N. nuda* individuals were run on 1% agarose gels in 1 × Tris/borate/EDTA (TBE) buffer at 1.5 V cm^−1^ for 1.5 h, stained with ethidium bromide, and visualized in an UV transilluminator (ST4 3026-WL/26M, Vilber Lourmat, Collégien, France) along with a 50 bp DNA Ladder (Thermo Fisher Scientific, Karlsruhe, Germany). Amplification of 7 out of 20 EST-SSR markers yielded fragments of the expected length (or longer) across the tested plants. These seven EST-SSR loci were used for further population genetic studies of *N. nuda* (Table 5).

#### 3.4.3. Genomic Microsatellite Markers

To broaden the SSR genotyping, we tested 12 microsatellite markers reported by [91] for *Mentha* spp. that reportedly amplified DNA of *Nepeta* sp. After careful analysis of the amplification success and reproducibility, as well as of variation in alleles, two SSR markers were selected for further analysis: Cont028-gene0.2 and Cont039-gene0.9 (Table 5).

#### 3.4.4. Amplification of the Selected Microsatellite Loci, Fragment Analysis, and Data Processing

The final PCR amplification of 9 selected molecular markers (7 EST-SSRs and 2 SSRs) was performed using 0.2 µM forward and reverse primers (Invitrogen, UK), the former being 5′-labeled with one of the four fluorescent dyes (Table 5). The PCR reaction program was the same as for the amplification optimization. PCR reactions were performed without multiplexing.

Two runs per individual were prepared for fragment analysis, each containing 4 or 5 markers (Table 5). Fragment analysis was performed on an ABI3730xl DNA Analyzer (Thermo Fisher Scientific, Foster City, CA, USA) using GeneScan 500 LIZ size standard. Data scoring was performed using Peak Scanner (Thermo Fisher Scientific, Foster City, CA, USA). The following parameters were determined for each population: percentage of polymorphic loci, the number of different alleles, the number of effective alleles, the number of private alleles, Shannon’s informative index, observed heterozygosity, expected heterozygosity, unbiased expected heterozygosity, and fixation index. To determine the sources of genetic variation within and between populations, AMOVA was performed using Wright’s F statistics [81,92]. Pairwise FST values between populations were also calculated [81]. Visualization of populations’ grouping was made through PCoA, using codominant genotypic distances among individuals. Population genetic analyses, AMOVA, and PCoA plotting were made by employing the GenAlEx 6.5 platform [93].

A mantel test between the genetic and geographic distances among the populations was performed using the IBD (Isolation By Distance) software [94] with 1000 randomizations. Population structuring was assessed using STRUCTURE 2.3.4 [95,96] with a burn-in length of 10,000 and a Markov Chain Monte Carlo (MCMC) of 100,000 randomizations, while the STRUCTURE HARVESTER online platform [97], which calculates the highest value of the second-order rate of change (DeltaK) according to the method of [98], was used to detect the number of K groups that best fits the dataset.

## 4. Conclusions

Based on previous studies dealing with the chemical characterization of different *N. nuda* accessions, it can be concluded that high intraspecific chemodiversity exists in this taxon. One must bear in mind that such implications might arise from different environmental conditions, growth stages of plants, sampling and extraction procedures, analytical techniques, and groups of metabolites analyzed. Literature data contain no comprehensive studies dealing with either inter- or intra-population variability of *N. nuda* aimed at determining sources of chemical diversity and providing explanations of this plasticity from the perspective of eco-evolutionary dynamics. The present study is the first attempt to explain variation in both metabolite profiles and genetic background within eleven populations of *N. nuda* from the Central Balkans. The low level of inter-population chemodiversity implies that differential environmental conditions might be less important for shaping the metabolomes of *N. nuda* populations, while their genetic backgrounds are of essential significance. This, at least, is true for the limited geographical area covered by the present study; this picture might vary if the whole species’ area was studied.

As well as improving our comprehension of both the genetic and the chemical diversity of indigenous populations of this species, within- and among-population variability was assessed to support and to facilitate germplasm utilization. The recorded multidimensional genetic and chemical diversity of *N. nuda* represents a source of variance, which was harnessed in the quest to develop high-resolution chemical markers for a mass-spectrometry-guided selection of productive genotypes. Screening the distribution of well-defined bioactive compounds belonging to the groups of phenolics (e.g., rosmarinic acid) and iridoids (both aglycones and glycosylated forms), within and among *N. nuda* populations, provides a tool for the selection of elite representatives of practical importance. Furthermore, the developed chemical markers may be of importance when exploring intra-individual chemodiversity, which may further help in deciphering the tissue- and organ- specific, as well as the developmentally regulated, patterns of changes in metabolic fingerprints of *Nepeta* taxa, as influenced by endogenous (e.g., phytohormones) and exogenous (environmental) factors. The reconstruction of metabolic networks and the adequate definition of the role of different metabolic classes in the defense strategy of plants, as well as the explanation of their mutual effects, will inform out background knowledge of the factors that shape the metabolomes of plants and thus of their adaptive success in changing environments. Finally, the gathered knowledge on *N. nuda* chemical variation within the Central Balkans will help us to explain the molecular background of this diversity and the regulatory mechanisms involved, which is the course of our future work.

## Figures and Tables

**Table 1:** UHPLC-ESI-QTOF-MS data on metabolites identified in leaf extracts of eleven *Nepeta nuda* populations.

No	Compound Name	*t*_R_, min	Molecular Formula, [M ± H] ^±^	Calculated Mass, *m*/*z*	Exact Mass, *m*/*z*	Δ mDa	MS^2^ Fragments, (% Base Peak)	Previously Reported in *Nepeta* sp.
***Hydroxybenzoic acid derivatives***								
**1**	**Galloyl hexoside**	1.41	C_13_H_15_O_10_^−^	331.06655	331.07343	−6.88	124.01732(17), 125.02673(94), **168.00944**(100), 169.01204(14), 313.05871(7)	[21]
**2**	**Gallic acid**	1.47	C_7_H_5_O_5_^−^	169.01370	169.02035	−6.65	107.01403(14), **125.02441**(100)	[22]
**3**	**Dihydroxybenzoic acid**	2.15	C_7_H_5_O_4_^−^	153.01933	153.02445	−5.12	**108.02182**(100), 109.02969(84)	[20]
**4**	**Dihydroxybenzoic acid hexoside**	2.42	C_13_H_15_O_9_^−^	315.07216	315.07717	−5.01	**108.02285**(100), 123.04534(9), 152.01279(66)	[19]
**5**	**Vanillic acid hexoside**	3.23	C_14_H_17_O_9_^−^	329.08781	329.09059	−2.79	**108.02225**(100), 123.04585(40), 152.01204(67), 167.03435(36)	No
**6**	**Hydroxybenzoic acid**	3.77	C_7_H_5_O_3_^−^	137.02390	137.02642	−2.52	NA	[20]
**7**	**Dihydroxybenzoic acid hexoside isomer**	4.65	C_13_H_15_O_9_^−^	315.07216	315.07757	−5.42	**109.03019**(100), 135.00806(5), 153.02023(29)	[19]
**8**	**Dihydroxybenzoic acid isomer**	5.35	C_7_H_5_O_4_^−^	153.01933	153.02154	−2.21	**109.02945**(100), 135.00889(22)	[20]
**9**	**Vanillic acid**	5.38	C_8_H_9_O_4_^+^	169.05010	169.05302	−2.92	**111.00905**(100), 126.03239(21), 141.05569(10)	[23]
**10**	**Hydroxybenzoic acid hexoside**	5.86	C_13_H_15_O_8_^−^	299.07670	299.08413	−7.43	**137.02614**(100)	[19]
***Hydroxycinnamic acid derivatives***								
**11**	**Syringic acid hexoside**	2.45	C_15_H_19_O_10_^−^	359.09837	359.10244	−4.07	123.04584(29), 135.04582(42), 179.03633(84), **197.04727**(100)	[19]
**12**	**Caffeoyltartaric acid**	3.77	C_13_H_11_O_9_^−^	311.04030	311.05026	−9.96	**135.04587**(100), 149.00992(14), 179.03556(17)	[24]
**13**	**Caffeoyltartronic acid**	4.31	C_12_H_9_O_8_^−^	281.03029	281.03386	−3.57	**109.03139**(100), 133.03049(4), 135.04562(3), 149.06061(3), 161.02493(4)	[25]
**14**	**Caffeic acid hexoside**	4.71	C_15_H_17_O_9_^−^	341.08739	341.09316	−5.77	133.03004(22), 135.04525(13), **161.02543**(100), 179.03550(8)	[19]
**15**	**Caffeoylglycolic acid**	4.84	C_11_H_9_O_6_^−^	237.04046	237.04355	−3.09	**133.03031**(100), 149.06131(6), 161.02503(51)	[26]
**16**	**Umbelliferone**	5.45	C_9_H_7_O_3_^+^	163.03897	163.04213	−3.16	103.05416(4), 107.04999(20), **117.03475**(100), 135.04366(28)	[27]
**17**	**Caffeoyltartronic acid isomer**	5.46	C_12_H_9_O_8_^−^	281.03029	281.03442	−4.13	**109.02964**(100), 135.04576(3), 149.06090(3)	[25]
**18**	**Sinapic acid**	6.00	C_11_H_11_O_5_^−^	223.06120	223.06334	−2.14	119.99436(15), 121.04802(18), **135.04597**(100), 145.05773(16), 151.01879(20)	[19]
**19**	**Caffeic acid hexoside isomer**	6.13	C_15_H_17_O_9_^−^	341.08739	341.09375	−6.36	**135.04617**(100), 149.06111(4), 161.02557(22), 177.05660(10), 179.03657(73)	[19]
**20**	**Dihydrocaffeic acid hexoside**	6.20	C_15_H_19_O_9_^−^	343.10294	343.10824	−5.30	119.05022(6), 137.06134(68), **181.05186**(100)	[28]
**21**	**Caffeic acid**	6.33	C_9_H_7_O_4_^−^	179.03440	179.03911	−4.71	107.05033(9), 117.03491(7), **135.04619**(100)	[19]
**22**	**Feruloyltartaric acid**	6.39	C_14_H_13_O_9_^−^	325.05606	325.06166	−5.60	119.05055(58), **134.03769**(100), 149.02713(7), 163.04084(14), 178.02764(4), 193.05174(13)	[29]
**23**	**Ethyl caffeate**	6.90	C_11_H_11_O_4_^−^	207.06628	207.06817	−1.88	**135.04752**(100), 163.04087(8)	[28]
**24**	**Caffeoylmalic acid**	6.95	C_13_H_11_O_8_^−^	295.04540	295.05022	−4.82	**135.04607**(100), 163.04099(12)	[24]
**25**	**Feruloylmalic acid**	7.89	C_14_H_13_O_8_^−^	309.06100	309.06740	−6.40	117.03496(12), **134.03810**(100), 147.03008(7), 178.02696(6), 193.05085(8)	No
**26**	**Rosmarinic acid**	8.56	C_18_H_15_O_8_^−^	359.07671	359.08532	−8.61	123.04620(13), 133.03059(17), 135.04630(27), **161.02576**(100), 179.03672(26), 197.04684(13)	[19]
**27**	**Ferulic acid**	8.63	C_10_H_9_O_4_^−^	193.05063	193.05230	−1.66	**133.03042**(100), 161.02513(14)	[30]
**28**	**Clinopodic acid A**	9.06	C_18_H_15_O_7_^−^	343.08180	343.08948	−7.68	133.03032(32), 135.04585(53), 145.03024(22), **161.02547**(100), 163.04039(37), 179.03673(11)	[19]
**29**	**Nepetoidin A or B**	10.31	C_17_H_13_O_6_^−^	313.07124	313.07606	−4.82	123.04611(10), 133.03063(55), 151.04076(12), **161.02620**(100)	[19]
***Iridoid glycosides***								
**30**	**Aucubin**	5.73	C_15_H_21_O_9_^−^	345.11911	345.12296	−3.85	101.02488(58), 113.02496(49), **119.04784**(100), 137.06027(11), 183.06797(3)	[31]
**31**	**6α-Hydroxyadoxoside**	5.79	C_17_H_25_O_11_^−^	405.14024	405.14119	−0.95	153.05597(6), 175.02649(5), 179.07328(7), **197.08320**(100)	[32]
**32**	**Nepetanudoside C *^a^***	6.13	C_17_H_23_O_10_^−^	387.12967	387.12974	−0.07	101.02413(94), 113.02500(94), 146.03664(60), **161.05728**(100), 165.05628(21), 179.07016(50)	[33]
**33**	**5-Deoxylamiol**	6.47	C_16_H_25_O_9_^−^	361.15041	361.15130	−0.89	101.02516(19), 115.04080(10), 119.08233(16), **137.09782**(100), 181.08803(11), 199.09816(18)	No
**34**	**1,5,9-*epi*-Deoxyloganic acid hexoside *^a^***	6.61	C_23_H_35_O_16_^−^	567.19257	567.19962	−7.05	109.06523(5), 135.08222(15), 153.09337(54), 197.08377(90), 239.09320(5), **359.13759**(100)	[34]
**35**	**Nepetanudoside A *^a^***	7.01	C_18_H_27_O_12_^−^	435.15080	435.15845	−7.65	**101.02477**(100), 183.06744(5), 227.09479(25)	[33]
**36**	**1,5,9-*epi*-Deoxyloganic acid**	7.41	C_16_H_23_O_9_^−^	359.13476	359.13819	−3.43	109.06682(9), **135.08297**(100), 153.09284(26), 197.08194(5)	[19]
**37**	**Nepetaside**	7.74	C_16_H_25_O_8_^−^	345.15549	345.15841	−2.92	**101.02493**(100), 113.02503(75), 119.03541(32), 167.10828(32), 185.11870(51)	[35]
**38**	**6-Deoxylamioside**	7.82	C_18_H_27_O_10_^−^	403.16097	403.16099	−0.01	179.10829(93), **197.11859**(100), 223.09835(52), 241.10981(27)	[19]
***Iridoid aglycones***								
**39**	**Genipin**	6.06	C_11_H_15_O_5_^+^	227.09190	227.09621	−4.31	**103.05515**(100), 121.06514(82), 131.05040(42), 167.07006(50)	No
**40**	**5,9-Dehydronepetalactone**	6.46	C_10_H_13_O_2_^+^	165.09101	165.09134	−0.33	103.05508(79), **105.06905**(100), 107.07625(61), 109.07648(13), 119.09073(70), 122.06183(18)	[36]
**41**	**Nepetaside aglycone acetate**	6.87	C_12_H_19_O_4_^+^	227.12830	227.13202	−3.72	105.07057(80), **107.08021**(100), 121.08498(48), 131.08589(93), 145.10006(26), 149.09439(53)	[35]
**42**	**Loganetin**	7.01	C_11_H_17_O_5_^+^	229.10705	229.11138	−4.33	**105.07101**(100), 115.05545(16), 133.06566(50), 161.06027(14)	No
**43**	**Deoxygeniposide aglycone**	7.01	C_11_H_15_O_4_^+^	211.09700	211.10148	−4.48	103.05506(23), **105.07104**(100), 115.05543(13), 133.06522(33), 135.08059(25), 161.06026(11)	[33]
**44**	**Nepetalactol**	7.28	C_10_H_17_O_2_^+^	169.12231	169.12247	−0.17	**105.07079**(100), 109.08535(7), 115.03444(14), 117.07101(11), 119.08376(10)	[30]
**45**	**7-Deoxyloganetic acid**	7.34	C_10_H_15_O_4_^+^	199.09649	199.09970	−3.21	105.07036(75), 107.08064(88), 111.07725(25), **115.05532**(100), 135.08059(58), 163.07571(22)	[37]
**46**	**Nepetaracemoside B aglycone**	7.35	C_10_H_13_O_3_^+^	181.08650	181.09269	−6.19	**105.06972**(100), 107.07002(44), 115.05538(67), 123.07979(61), 125.04424(16), 151.03903(63)	[38]
**47**	***trans*,*trans*-Nepetalactone**	7.61	C_10_H_15_O_2_^+^	167.10666	167.10699	−0.34	103.03409(23), **105.07013**(100, 107.08300(55), 115.05804(16), 123.11236(43), 134.06489(15)	[30]
**48**	**De-4-methylnepetalactol**	8.76	C_9_H_15_O_2_^+^	155.10666	155.10792	−1.26	107.08620(11), **109.10239**(100)	[39]
**49**	**7-Deoxyloganetin**	9.06	C_11_H_15_O_4_^−^	211.09700	211.10073	−3.73	**101.02417**(100), 107.03404(13), 109.07041(16), 121.07518(8), 123.06118(15)	[37]
**50**	**Nepetalic acid**	9.61	C_10_H_15_O_3_^−^	183.10267	183.10321	−0.54	107.05100(92), 121.06682(42), 135.08171(27), **137.09772**(100), 165.09377(41)	[40]
**51**	**Dihydronepetalactone**	10.92	C_10_H_17_O_2_^+^	169.12231	169.12267	−0.36	105.07085(11), 107.08429(8), 109.10045(30), 121.10647(8), **123.11750**(100)	[41]
**52**	***cis*,*trans*-Nepetalactone**	11.18	C_10_H_15_O_2_^+^	167.10666	167.10818	−1.52	105.07099(10), **111.04573**(100), 121.09798(3)	[30]
**53**	**Nepetalactol acetate**	11.19	C_12_H_19_O_3_^+^	211.13287	211.13652	−3.65	**105.07029**(100), 107.08509(47), 119.08591(43), 125.03622(16), 128.03647(18), 133.08077(45)	[42]
**54**	***trans*,*cis*-Nepetalactone**	12.07	C_10_H_15_O_2_^+^	167.10666	167.10855	−1.89	105.07139(15), **111.04675**(100), 121.09868(5)	[30]
***Flavonoid glycosides***								
**55**	**Luteolin 7-*O*-(6″-hexosyl)-hexuronide**	5.59	C_27_H_27_O_17_^−^	623.12486	623.13153	−6.68	**109.03032**(100), 149.06083(4), 161.02436(11), 179.03545(3), 193.05110(20)	[24]
**56**	**Luteolin 7-*O*-(2″-hexuronyl)-hexuronide**	7.21	C_27_H_25_O_18_^−^	637.10412	637.11163	−7.51	113.02472(21), 175.02568(8), 193.03640(21), **285.04251**(100), 351.05935(77)	[27]
***Flavonoid aglycones***								
**57**	**Thymusin**	10.38	C_17_H_13_O_7_^−^	329.06615	329.07465	−8.50	117.03536(7), 151.00456(4), 179.00091(27), 271.0266(33), **299.02439**(100), 314.04460(7)	[19]
**58**	**Chrysoeriol**	11.06	C_16_H_11_O_6_^−^	299.05569	299.06267	−6.98	133.02977(6), 151.00393(3), 256.03830(7), **284.03484**(100)	[43]
**59**	**Cirsimaritin**	11.19	C_17_H_13_O_6_^−^	313.07124	313.07807	−6.83	163.00548(13), 255.03267(19), 269.04769(6), **283.02700**(100), 297.04336(15), 298.04845(8)	[19]
**60**	**Xanthomicrol**	11.39	C_18_H_17_O_7_^+^	345.09745	345.10480	−7.35	148.05295(7), 269.04548(9), **284.07100**(100), 312.06655(81), 315.05252(7), 330.07592(48)	[19]
**61**	**Acacetin**	11.93	C_16_H_11_O_5_^−^	283.06067	283.06701	−6.34	117.03558(8), 151.00393(5), 239.03599(6), **268.04163**(100)	[19]
**62**	**Salvigenin**	13.14	C_18_H_17_O_6_^+^	329.10250	329.10925	−6.75	133.06707(13), 240.07974(10), **268.07475**(100), 296.06940(78), 314.07896(22), 329.10246(24)	[44]
***Other metabolites***								
**63**	**Quinic acid**	0.80	C_6_H_7_O_7_^−^	191.01920	191.02455	−5.35	**111.00978**(100)	[19]
**64**	**3-(3,4-Dihydroxyphenyl)-lactic acid**	1.98	C_9_H_9_O_5_^−^	197.04555	197.04603	−0.48	107.04994(12), 109.03039(17), 117.03462(7), 123.04566(93), **135.04557**(100)	[45]
**65**	**Hexenyl-pentosyl-hexoside**	2.09	C_17_H_29_O_10_^−^	393.17662	393.18045	−3.83	101.02318(23), 113.02680(26), 119.01591(12), 123.02787(3), **131.03518**(100), 161.04879(5)	[32]
**66**	**Schizonepetin**	6.46	C_10_H_15_O_3_^+^	183.10157	183.10308	−1.51	105.07057(27), 107.08372(24), **109.10040**(100), 117.07044(13), 119.08775(34), 123.07086(10)	[46]
**67**	**12-*O*-Hexosyl-jasmonate**	6.87	C_18_H_27_O_9_^−^	387.16606	387.16637	−0.32	**101.02502**(100), 113.02505(72), 119.03558(42), 163.11382(51), 207.10349(81)	[19]
**68**	**Schizonepetin isomer**	7.48	C_10_H_15_O_3_^+^	183.10157	183.10547	−3.90	105.07086(100), 107.07648(92), 109.07756(52), 117.07182(45), 119.08400(48), 121.09486(54)	[46]
**69**	**2,5-Dimethyl-hexanedioic acid**	7.49	C_8_H_13_O_4_^−^	173.08193	173.08272	−0.79	**109.06619**(100), 111.08276(73), 129.09233(6)	[47]
**70**	**2-Carboxy-3-methyl-cyclopentaneacetic acid**	8.15	C_9_H_13_O_4_^−^	185.08140	185.08673	−5.33	117.02066(13), 121.04882(13), 123.06020(68), 123.08541(57), **141.09308**(100), 167.04390(13)	[48]
**71**	**Argolic acid A rhamnoside**	8.76	C_16_H_27_O_8_^−^	347.17114	347.17451	−3.37	101.02454(9), **139.11356**(100), 163.06222(17), 183.10372(88), 201.11441(82)	No
**72**	**2-Carboxy-α,3-dimethyl-cyclopentaneacetic acid**	9.03	C_10_H_15_O_4_^−^	199.09700	199.10188	−4.88	137.09790(29), **155.10907**(100)	[49]
**73**	**9-Oxononanoic acid**	9.12	C_9_H_15_O_3_^−^	171.10267	171.10333	−0.67	123.08108(6), **125.09811**(100)	[49]
**74**	**2-Carboxy-3-methyl-cyclopentaneacetic acid isomer**	9.23	C_9_H_13_O_4_^−^	185.08140	185.08669	−5.29	125.09854(33), **141.09333**(100)	[50]
**75**	**Argolic acid A**	9.30	C_10_H_17_O_4_^−^	201.11323	201.11437	−1.14	111.07898(26), 123.05859(5), 137.10383(28), **139.11271**(100), 183.10100(35)	[51]
**76**	**Argolic acid A methyl ether rhamnoside**	9.37	C_17_H_29_O_8_^−^	361.18679	361.19035	−3.56	153.12884(41), 163.06281(16), **197.11928**(100), 215.13001(52)	No
**77**	**Nepetonic acid**	10.10	C_9_H_13_O_3_^−^	169.08702	169.08904	−2.02	123.08205(15), **125.09862**(100), 151.07836(21)	[32]
**78**	**2-Carboxy-α,3-dimethyl-cyclopentaneacetic acid dimethyl ester**	10.45	C_12_H_19_O_4_^−^	227.12888	227.12899	−0.11	111.06175(16), 165.12273(11), **183.14644**(100)	[41]

*^a^* Formic acid adduct; *t*_R_—retention time (min); Δ mDa—mean mass accuracy; NA—not available. The [M ± H] ^±^ column indicates in which ionization mode the corresponding compound was identified. Dominant fragments are represented boldfaced in the column MS^2^ fragments.

**Table 2:** GC/MS characterization of aboveground extracts of 11 *Nepeta nuda* populations from the Central Balkans (relative content, %).

No.	RT	Assignment	Molecular Formula	Accession										
				Židilje	Straža	Vinatovača	Debeli Lug	Gornje Selo	Brodice	Selište	Rtanj	Janjska Reka	Vlasina	Donji Krivodol
***Monoterpene hydrocarbons***				**5.97**	**7.59**	**6.74**	**6.56**	**5.41**	**13.38**	**10.78**	**13.17**	**3.45**	**3.70**	**6.04**
**1**	6.78	*α*-Pinene ^a^	C_10_H_16_	0.29	2.65	1.68	2.10	/	3.33	2.91	2.33	/	/	1.41
**2**	7.26	Sabinene	C_10_H_16_	/	/	1.04	/	0.94	1.84	1.59	1.20	2.89	0.57	0.94
**3**	7.30	*β*-Pinene ^a^	C_10_H_16_	4.93	4.04	3.56	3.93	3.63	6.15	5.45	4.08	/	2.09	3.20
**4**	7.41	*β*-Myrcene	C_10_H_16_	0.75	0.61	0.46	0.53	0.40	0.88	0.83	4.27	/	0.75	0.49
**5**	7.81	o-Cymene	C_10_H_14_	/	0.29	/	/	0.44	0.50	/	0.63	0.56	0.29	/
**6**	7.87	D-Limonene ^a^	C_10_H_16_	/	/	/	/	/	0.68	/	0.66	/	/	/
***Oxygenated monoterpenes***				**44.60**	**31.27**	**27.07**	**28.53**	**40.12**	**31.13**	**28.65**	**32.26**	**23.00**	**37.00**	**19.10**
**7**	7.90	1,8-Cineole	C_10_H_18_O	37.23	24.63	24.47	25.14	39.19	29.78	27.22	27.91	20.89	21.11	17.86
**8**	9.21	*δ*-Terpineol	C_10_H_18_O	/	0.39	/	0.38	/	/	/	0.45	0.30	0.25	/
**9**	9.42	*α*-Terpineol	C_10_H_18_O	0.54	1.64	0.78	1.69	0.82	0.63	1.21	1.81	1.35	0.94	0.92
**10**	9.48	*p*-Mentha-1,5-dien-7-ol	C_10_H_16_O	/	0.27	/	0.35	/	/	/	1.81	0.29	/	/
**11**	10.86	*trans*, *trans*-Nepetalactone	C_10_H_14_O_2_	6.83	3.06	1.57	0.97	/	/	0.13	/	0.17	1.17	0.23
**12**	10.92	*cis*, *trans*-Nepetalactone ^a^	C_10_H_14_O_2_	/	1.28	0.25	/	0.11	0.72	0.09	0.28	/	13.53	0.09
***Sesquiterpene hydrocarbons***				**43.49**	**54.17**	**59.71**	**54.13**	**39.76**	**46.6**	**50.65**	**26.94**	**59.61**	**44.93**	**69.84**
**13**	10.89	*α*-Cubebene	C_15_H_24_	/	1.28	0.80	/	/	/	0.60	/	0.73	/	0.90
**14**	10.96	*α*-Copaene	C_15_H_24_	0.72	0.90	2.63	0.43	0.54	0.46	/	/	/	/	/
**15**	11.06	(-)-*β*-Bourbonene	C_15_H_24_	2.59	3.43	/	/	3.13	/	/	3.19	3.28	/	4.91
**16**	11.25	alpha-Gurujene	C_15_H_24_	/	/	/	/	/	/	/	/	0.43	/	0.58
**17**	11.33	beta-Caryophyllene ^a^	C_15_H_24_	12.09	17.24	9.99	12.60	8.20	14.42	9.12	11.00	16.90	10.78	16.56
**18**	11.39	*β-Copaene*	C_15_H_24_	/	0.69	0.56	0.60	0.47	/	1.08	/	0.73	1.06	1.03
**19**	11.41	Bicyclosesquiphellandrene	C_15_H_24_	/	/	/	/	/	0.53	0.62	/	/	/	/
**20**	11.45	*cis*-*β*-Farnesene	C_15_H_24_	1.11	1.29	0.47	0.53	7.01	1.06	/	/	1.06	2.52	/
**21**	11.50	Isogermacrene D	C_15_H_24_	/	0.44	/	/	/	/	/	/	/	0.37	0.66
**22**	11.58	*α*-Humulene	C_15_H_24_	2.67	3.77	2.53	3.28	2.44	3.87	2.35	3.13	4.38	2.60	4.30
**23**	11.76	Germacrene D ^a^	C_15_H_24_	20.71	18.85	37.50	31.68	12.32	21.84	33.80	5.76	26.73	21.40	35.45
**24**	11.84	*β*-Bisabolene	C_15_H_24_	3.09	4.04	2.01	2.40	2.53	3.33	/	2.35	1.67	3.13	/
**25**	11.87	Bicylogermacrene	C_15_H_24_	/	/	/	/	/	/	/	/	1.64	/	3.21
**26**	11.99	Cadina-3,5-diene	C_15_H_24_	0.51	2.24	2.83	2.02	1.42	0.00	2.69	1.51	0.00	2.68	1.85
**27**	12.03	*β*-Cadinene	C_15_H_24_	/	/	/	/	/	1.09	/	/	1.24	/	/
**28**	12.29	Patchoulane	C_15_H_26_	/	/	0.39	0.59	1.70	/	0.39	/	0.82	0.39	0.39
***Oxygenated sesquiterpenes***				**1.22**	**2.71**	**2.01**	**4.03**	**9.47**	**3.6**	**1.56**	**12.25**	**6.99**	**2.64**	**2.7**
**29**	12.51	Caryophyllene oxide	C_15_H_24_O	1.22	2.44	2.01	3.47	8.88	3.22	1.56	12.25	6.25	2.64	2.30
**30**	12.68	Humulene epoxide I	C_15_H_24_O	/	0.27	/	0.56	0.59	0.38	/	/	0.74	/	0.40
**Total**				95.28	95.74	95.53	93.25	94.76	94.71	91.64	84.62	93.05	88.27	97.68
**EO yield (µL/g DW)**				**5.68**	**5.37**	**5.08**	**4.62**	**5.36**	**4.36**	**5.02**	**2.19**	**4.53**	**5.97**	**5.72**

Abbreviations: /—not found; DW—dry weight; RT—retention time; ^a^—confirmed by the authentic standard.

**Table 3:** Localities of collection of *Nepeta nuda* material used in the study. The number of individuals per population used for the population genetics analysis and the yield of essential oils extracted from plants of the specific populations are also given (% PL—percentage of polymorphic loci; Na—mean number of alleles in a population; Ne—number of effective alleles; PA—number of private alleles; I—Shannon’s informative index; Ho—observed heterozygosity; He—expected heterozygosity; uHe—unbiased expected heterozygosity; F—fixation index.).

Population	% PL	Na	Ne	PA	I	Ho	He	uHe	F
**Selište**	88.89	2.222 ± 0.324	1.769 ± 0.273	1	0.653 ± 0.112	0.521 ± 0.148	0.413 ± 0.070	0.450 ± 0.076	−0.133 ± 0.250
**Brodica**	77.78	2.333 ± 0.333	1.770 ± 0.245	0	0.593 ± 0.137	0.489 ± 0.111	0.358 ± 0.077	0.386 ± 0.084	−0.362 ± 0.068
**Debeli Lug**	100	2.444 ± 0.242	1.864 ± 0.226	0	0.676 ± 0.102	0.394 ± 0.078	0.411 ± 0.056	0.455 ± 0.064	0.050 ± 0.162
**Rtanj**	88.89	2.556 ± 0.338	2.131 ± 0.249	0	0.755 ± 0.139	0.552 ± 0.113	0.462 ± 0.078	0.506 ± 0.086	−0.178 ± 0.118
**Straža**	88.89	3.111 ± 0.588	1.969 ± 0.298	0	0.746 ± 0.160	0.411 ± 0.093	0.408 ± 0.077	0.430 ± 0.081	−0.006 ± 0.115
**Vinatovača**	77.78	3.333 ± 0.667	2.445 ± 0.502	2	0.836 ± 0.209	0.478 ± 0.109	0.446 ± 0.100	0.469 ± 0.106	−0.073 ± 0.088
**D. Krivodol**	88.89	2.333 ± 0.333	1.856 ± 0.298	1	0.683 ± 0.126	0.501 ± 0.101	0.424 ± 0.078	0.448 ± 0.082	−0.199 ± 0.117
**Janjska reka**	88.89	2.667 ± 0.471	1.839 ± 0.232	2	0.658 ± 0.130	0.467 ± 0.103	0.394 ± 0.068	0.415 ± 0.072	−0.160 ± 0.122
**Židilje**	100	3.444 ± 0.669	2.556 ± 0.518	1	0.901 ± 0.187	0.544 ± 0.091	0.488 ± 0.086	0.513 ± 0.090	−0.134 ± 0.063
**Gornje Selo**	100	3.222 ± 0.364	2.430 ± 0.291	2	0.931 ± 0.136	0.517 ± 0.097	0.524 ± 0.070	0.589 ± 0.079	−0.001 ± 0.118
**Vlasina**	100	3.444 ± 0.412	2.255 ± 0.312	3	0.897 ± 0.117	0.523 ± 0.062	0.504 ± 0.051	0.536 ± 0.056	−0.059 ± 0.090

**Table 4:** Localities of collection of *Nepeta nuda* material used in the study. The number of individuals per population used for the population genetics analysis and the yield of essential oils extracted from plants of the specific populations are also given.

Locality	Coordinates	Elevation [m]	Date of collection	Voucher No.	No. of Individuals Used for Microsatellite Analysis
**Židilje**	44°00′42″ N; 21°38′45″ E	580	13 July 2022		10
**Straža**	43°50′46″ N; 21°42′03″ E	535	13 July 2022	17850	10
**Vinatovača**	44°04′14″ N; 21°45′36″ E	638	14 July 2022	17849	10
**Debeli Lug**	44°21′45″ N; 21°54′01″ E	332	7 July 2022	17854	7
**Gornje selo**	42°11′21″ N; 20°56′20″ E	1722	11 July 2022		5
**Brodica**	44°29′04″ N; 21°50′27″ E	237	7 July 2022	17848	7
**Selište**	43°33′33″ N; 20°49′56″ E	1055	21 June 2022	17847	7
**Rtanj**	43°44′13″ N; 21°57′03″ E	675	13 July 2022		6
**Janjska reka**	43°25′33″ N; 22°31′11″ E	587	21 July 2022	17853	10
**Vlasina**	42°41′39″ N; 22°22′44″ E	1332	14 July 2022	17846	10
**Donji Krivodol**	43°06′18″ N; 22°55′41″ E	811	20 July 2022	17851	10

**Table 5:** Characteristics of nine microsatellite markers used for assessing the population structure of eleven populations of *N. nuda*. The expected sizes of the fragments (in bp) and their size ranges are given for a sample of 92 individuals. Ta = annealing temperature.

Locus	Repeat Motif	F and R Primer Sequences (5′-3′)	5′-Modification	Ta [°C]	Expected Length [bp]	Observed Size Range [bp]
**MN03 ^1^**	GGA	CGTCAAGTACTTTGAGAAGGA	6-FAM	55	168	162–180
		CTACTTTCCACCTCCGGTA				
**MN22 ^1^**	CTG	GTGGGGATTAATCTCAATGAT	6-FAM	55	128	118–127
		AGGAACGAACAACAATCAATA				
**MN26 ^2^**	CTG	CCCAACTATCCTTCATCTACC	6-FAM	55	151	445–507
		AGACGACGGACTTCCTTTAT				
**MN44 ^2^**	GAA	TATGAATTGGAGAAAGAGCTG	NED	52.5	150	148–157
		TGAAATGACCGTATGATTTTC				
**MN53 ^1^**	AGG	GTTGAGTTTCAACAAGACGAA	VIC	55	165	152–167
		CCGAGTTTCTTATCACATTCA				
**MN62 ^2^**	CAG	AGCCTCTTGTTCAAAACACTA	VIC	55	155	148–160
		CTTTTGTCTAACTGCAACGAT				
**MN74 ^2^**	TGA	TGAATGATTTCCTTCGTCTTA	6-FAM	55	140	133–159
		CCTGAATCAAAATGTAGGTGA				
**Cont028-gene0.2 ^1^**	(TC)_8_	AATAGGGAGTCTGCTGCTAGGT	NED	61	199	106–171
		CAGTGACTCCAACTCAACGGTATA				
**Cont039-gene0.9 ^2^**	(CT)_11_	ACATCTCCCGAATTATCTGTCCAT	PET	55	140	133–159
		GCTGTATAATACTTTGTGTTGATTGTCC				

^1^ Loci multiplexed in the first run of fragment analysis; ^2^ loci multiplexed in the second run of fragment analysis.

## Data Availability

The authors declare that the data supporting the findings of this study are available within the paper. Should any raw data files be needed in another format, they are available from the corresponding author upon reasonable request.

## Supplementary Materials

The following supporting information can be downloaded at https://www.mdpi.com/article/10.3390/plants13111483/s1: Figure S1: Base peak chromatograms, in negative and positive ionization modes, of 11 different *Nepeta nuda* leaf extracts; for peak annotation see retention times in Table 1 and peak areas from Table S2; Figure S2: A proposed fragmentation pathway of (**A**) compound **5** (vanillic acid hexoside) and (**B**) compound **25** (feruloylmalic acid); Figure S3: A proposed fragmentation pathway of compound **33** (5-deoxylamiol); Figure S4: A proposed fragmentation pathway of (**A**) compound **39** (genipin) and (**B**) compound **42** (loganetin); Figure S5: A proposed fragmentation pathway and MS^2^ spectra of (**A**) compound **71** (argolic acid A rhamnoside) and (**B**) compound **76** (argolic acid A methyl ether rhamnoside); Table S1: Review of the literature related to the detection of various compounds (iridoids, phenolics, and terpenes) in *Nepeta nuda* extracts [99,100,101,102,103,104,105,106]; Table S2: Peak areas of compounds identified using QToF MS in eleven different *Nepeta nuda* leaf extracts; Table S3: Validation parameters of the SRM method of the UHPLC/DAD/(±)HESI-MS^2^ for the quantification of targeted compounds.
