# Supplementary material for: Patterns of Genetic Variation of Nepeta nuda L. from the Central Balkans: Understanding Drivers of Chemical Diversity
Source: Plants (Basel). 2024 May 27;13(11):1483. doi: 10.3390/plants13111483 (PMC11174911; doi:10.3390/plants13111483)
Supplement: Supplementary file 1 [file plants-13-01483-s001.zip › plants-2993510-supplementary.pdf]

Supplementary material

Content

Table S1. Review of the literature related to the detection of various compounds (iridoids, phenolics, and terpenes) in *Nepeta nuda* extracts.

Table S2. Peak areas of compounds identified using QToF MS in eleven different *Nepeta nuda* leaf extracts.

Table S3. Validation parameters of the SRM method of the UHPLC/DAD/(±)HESI-MS^2 for the quantification of targeted compounds.

Figure S1. Base peak chromatograms, in negative and positive ionization modes, of eleven different *Nepeta nuda* leaf extracts; for peak annotation see retention times in Table 1 and peak areas from Table S2.

Figure S2. A proposed fragmentation pathway of **(A)** compound **5** (vanillic acid hexoside) and **(B)** compound **25** (feruloylmalic acid).

Figure S3. A proposed fragmentation pathway of compound **33** (5-deoxylamiol).

Figure S4. A proposed fragmentation pathway of **(A)** compound **39** (genipin) and **(B)** compound **42** (loganetin).

Figure S5. A proposed fragmentation pathway and MS^2 spectra of **(A)** compound **71** (argolic acid A rhamnoside) and **(B)** compound **76** (argolic acid A methyl ether rhamnoside).

Table S1. Review of the literature related to the detection of various compounds (iridoids, phenolics, and terpenes) in *Nepeta nuda* extracts.

Compound	Type of extract	References
Iridoids		
***cis*,*trans*-Nepetalactone**	EO	De Pooter et al., 1987 [61]; Kobaisy et al., 2005 104; Mancini et al., 2009 [99]; Narimani et al., 2017 [13]; Akbaba et al., 2021 [100]
	HE and AE	Kökdil et al., 1999 [40]
***trans*,*cis*-Nepetalactone**	EO	Regnier et al., 1967 [41]; De Pooter et al., 1987 [61]; Mancini et al., 2009 [99]; Gkinis et al., 2010 [14]; Bozok et al., 2017 [4]; Narimani et al., 2017 [13]; Akbaba et al., 2021 [100]; Aćimović et al., 2022 [62]
***cis*,*cis*-Nepetalactone**	EO	De Pooter et al., 1987 [61]; Kobaisy et al., 2005 [8]; Mancini et al., 2009 [99]; Bozari et al., 2013 [101]; Gormez et al., 2013 [6]
	HE and AE	Kökdil et al., 1999 [40]
	EA	Zaharieva et al., 2023 [11]
***trans*,*trans*-Nepetalactone**	EO	De Pooter et al., 1987 [61]; Handjieva et al., 1996 [57]
**Iridomyrmecin**	EO	Handjieva et al., 1996 [57]
**Isoiridomyrmecin**	EO	Handjieva et al., 1996 [57]
**Nepetalic acid**	HE	Kökdil et al., 1999 [40]
	EO	Handjieva et al., 1996 [57]
**Dehydronepetalactone**	EO	Handjieva et al., 1996 [57]
	HE and AE	Kökdil et al., 1999 [40]
**1,5,9-*epi*-Deoxyloganic acid**	HE and AE	Kökdil et al., 1999 [40]
	MeOH	Dienaitė et al., 2018 [27]; Petrova et al., 2022 [10]
**Epideoxyloganic acid hexoside**	MeOH	Petrova et al., 2022 [10]
**Epideoxyloganic acid pentoside**	MeOH	Petrova et al., 2022 [10]
**Geniposidic acid**	MeOH	Petrova et al., 2022 [10]
**Dihydrocornic acid**	MeOH	Petrova et al., 2022 [10]
**Secologanin**	MeOH	Petrova et al., 2022 [10]
**Verminoside**	MeOH	Smiljković et al., 2018 [15]
**Velpetin**	MeOH	Takeda et al., 1995 [33]
**Nepetanudoside A**	MeOH	Takeda et al., 1995 [33]
**Nepetanudoside B**	MeOH	Takeda et al., 1995 [33]
**Nepetanudoside C**	MeOH	Takeda et al., 1995 [33]
**Nepetanudoside D**	MeOH	Takeda et al., 1995 [33]
**Nepetanudone**	HE and AE	Kökdil et al., 1998b [102]
Phenolics		
**Genkwanin**	MeOH	Jamzad et al., 2003 [43]
**Isothymusin**	MeOH	Jamzad et al., 2003 [43]

**8-Hydroxysalvigenin**	MeOH	Jamzad et al., 2003 [43]
**4-Hydroxybenzoic acid**	WE and EtOH	Teber and Bursal, 2020 [63]
	MeOH	Petrova et al., 2022 [10]
**Acacetin**	MeOH	Mišić et al., 2015 [20]
**Aesculetin**	MeOH	Petrova et al., 2022 [10]
**Aesculin**	MeOH	Mišić et al., 2015 [20]; Petrova et al., 2022 [10]
**Apigenin**	WE and MeOH	Dienaitė et al., 2018 [27]
	WE and EtOH	Teber and Bursal, 2020 [63]
	MeOH	Mišić et al., 2015 [20]; Aras et al., 2016 [14]; Sarikurkcu et al., 2019 [5]; Petrova et al., 2022 [10]
**Apigenin 7-*O*-(acetyl) hexuronide**	MeOH	Petrova et al., 2022 [10]
**Apigenin 7-*O*-(acetyl-caffeoyl) hexuronide**	MeOH	Petrova et al., 2022 [10]
**Apigenin 7-*O*-(caffeoyl) hexuronide**	MeOH	Petrova et al., 2022 [10]
**Apigenin 7-*O*-(feruloyl) hexuronide**	MeOH	Petrova et al., 2022 [10]
**Apigenin 7-*O*-(sinapoyl) hexuronide**	MeOH	Petrova et al., 2022 [10]
**Apigenin 7-*O*-hexoside**	MeOH	Petrova et al., 2022 [10]
**Apigenin 7-*O*-hexuronide**	MeOH	Petrova et al., 2022
**Apigenin 7-*O*-hexuronide methyl ester**	MeOH	Petrova et al., 2022 [10]
**Astragalin**	MeOH	Mišić et al., 2015 [20]; Petrova et al., 2022 [10]
**Benzoic acid methyl ester**	WE and MeOH	Dienaitė et al., 2018 [27]
**Benzoyl tartaric acid**	MeOH	Petrova et al., 2022 [10]
**Caffeic acid**	WE	Hinkov et al., 2020 [103]
	WE and EtOH	Teber and Bursal, 2020 [63]
	WE and MeOH	Dienaitė et al., 2018 [27]
	MeOH	Mišić et al., 2015 [20]; Aras et al., 2016 [14]; Sarikurkcu et al., 2019 [5]; Petrova et al., 2022 [10]
**Caffeic acid hexoside**	MeOH	Petrova et al., 2022 [10]
**Caffeic acid hexuronide**	MeOH	Petrova et al., 2022 [10]
**Caffeolymalic acid**	WE and MeOH	Dienaitė et al., 2018 [27]
**Caffeoyltartaric acid**	MeOH	Petrova et al., 2022 [10]
**Calcelarioside A/B**	MeOH	Smiljković et al., 2018 [15]
**Chlorogenic acid**	WE	Hinkov et al., 2020 [103]
	WE and MeOH	Dienaitė et al., 2018 [27]
	MeOH	Mišić et al., 2015 [20]; Aras et al., 2016 [14]; Sarikurkcu et al., 2019 [5]; Petrova et al., 2022

		[10]
**Chrysin**	MeOH	Mišić et al., 2015 [20]
**Chrysoeriol**	WE	Hinkov et al., 2020 [103]
**Cinnamic acid**	MeOH	Mišić et al., 2015 [20]
	WE	Hinkov et al., 2020 [103]
**Cirsimaritin**	WE	Hinkov et al., 2020 [103]
	MeOH	Jamzad et al., 2003 [43]; Petrova et al., 2022 [10]
**Clinopodic acid A**	MeOH	Petrova et al., 2022 [10]
**Coumaric acid**	WE and EtOH	Teber and Bursal, 2020 [63]
	MeOH	Aras et al., 2016 [14]
**Dicaffeoylquinic acid**	MeOH	Mišić et al., 2015 [20]
**Dimetoxy cinnamic acid**	MeOH	Mišić et al., 2015 [20]
**Ethyl caffeate**	MeOH	Petrova et al., 2022 [10]
**Eryodictyol**	MeOH	Petrova et al., 2022 [10]
**Eukovoside**	MeOH	Smiljković et al., 2018 [15]
**Ferulic acid**	WE	Hinkov et al., 2020 [103]
	WE and MeOH	Dienaitė et al., 2018 [27]
	MeOH	Mišić et al., 2015 [20]; Sarikurkcu et al., 2019 [5]; Petrova et al., 2022 [10]
**Feruloyltartaric acid**	MeOH	Petrova et al., 2022 [10]
**Galangin**	MeOH	Mišić et al., 2015 [20]
**Gallic acid**	WE	Hinkov et al., 2020 [103]
	MeOH	Petrova et al., 2022 [10]
**Gallic acid hexoside**	MeOH	Petrova et al., 2022 [10]
**Gentisic acid**	MeOH	Petrova et al., 2022 [10]
**Hispidulin**	MeOH	Petrova et al., 2022 [10]
**Hydroxybenzoic acid hexoside**	MeOH	Mišić et al., 2015 [20]; Petrova et al., 2022 [10]
**Isorhamentin**	MeOH	Petrova et al., 2022 [10]
**Kaempferol**	WE and EtOH	Teber and Bursal, 2020 [63]
	MeOH	Aras et al., 2016 [14]
**Kaempferol dimethyl ether**	MeOH	Mišić et al., 2015 [20]
**Kaempferol monomethyl ether**	MeOH	Mišić et al., 2015 [20]
**Ligstroside hexoside**	MeOH	Smiljković et al., 2018 [15]
**Lithospermic acid A**	MeOH	Smiljković et al., 2018 [15]
**Luteolin**	WE and MeOH	Dienaitė et al., 2018
	MeOH	Mišić et al., 2015 [20]; Aras et al., 2016 [14]
**Luteolin 7-*O*-diglucuronide**	WE and MeOH	Dienaitė et al., 2018 [27]
**Luteolin 7-*O*-(feruloyl) hexuronide**	MeOH	Petrova et al., 2022 [10]
**Luteolin 7-*O*-hexuronide**	MeOH	Petrova et al., 2022 [10]
**Luteolin 7-*O*-(caffeoyl) hexuronide**	MeOH	Petrova et al., 2022 [10]

**Luteolin 7-*O*-(acetyl) hexuronide**	MeOH	Petrova et al., 2022 [10]
**Methyl rosmanirate**	MeOH	Petrova et al., 2022 [10]
**Methyl salvianolate**	MeOH	Petrova et al., 2022 [10]
**Myricetin**	WE and EtOH	Teber and Bursal, 2020 [63]
**Nepetoidin B**	MeOH	Petrova et al., 2022 [10]
**Naringenin**	MeOH	Mišić et al., 2015 [20]; Petrova et al., 2022 [10]
**Plantamajoside**	MeOH	Smiljković et al., 2018 [15]
**Plantamajoside hexoside**	MeOH	Smiljković et al., 2018 [15]
**Protocatechuic acid**	WE and EtOH	Teber and Bursal, 2020 [63]
	WE	Hinkov et al., 2020 [103]
	MeOH	Mišić et al., 2015 [20]; Smiljković et al., 2018 [15]; Sarikurkcu et al., 2019 [5]; Petrova et al., 2022 [10]
**Quercetin**	WE and EtOH	Teber and Bursal, 2020 [63]
	WE	Hinkov et al., 2020 [103]
	MeOH	Mišić et al., 2015 [20]
**Quercetin dimethyl ether**	MeOH	Mišić et al., 2015 [20]
**Quercetin trimethyl ether**	MeOH	Mišić et al., 2015 [20]
**Quercetin tetramethyl ether**	MeOH	Mišić et al., 2015 [20]
**Rhamnetin**	MeOH	Aras et al., 2016 [14]
**Rosmarinic acid**	WE and MeOH	Dienaitė et al., 2018 [27]
	WE	Hinkov et al., 2020 [103]
	MeOH	Mišić et al., 2015 [20]; Aras et al., 2016 [14]; Smiljković et al., 2018 [15]; Petrova et al., 2022 [10]
**Rutin**	WE	Hinkov et al., 2020 [103]
	MeOH	Petrova et al., 2022 [10]
**Salvianolic acid A**	MeOH	Smiljković et al., 2018 [15]
**Salvianolic acid B**	MeOH	Smiljković et al., 2018 [15]
**Salvianolic acid C**	MeOH	Petrova et al., 2022 [10]
**Syringic acid**	WE and MeOH	Dienaitė et al., 2018 [27]
	MeOH	Mišić et al., 2015 [20]; Petrova et al., 2022 [10]
**Syringic acid hexoside**	MeOH	Mišić et al., 2015 [20]
**Thymusin**	MeOH	Petrova et al., 2022 [10]
**Umbelliferone**	WE and MeOH	Dienaitė et al., 2018 [27]
**Vanillic acid**	WEs	Hinkov et al., 2020 [103]
	MeOH	Mišić et al., 2015 [20]; Petrova et al., 2022 [10]
**Vanillin**	MeOH	Petrova et al., 2022 [10]
**Xanthomicrol**	MeOH	Petrova et al., 2022 [10]
Other monoterpenes		

**1,8-Cineole (eucalyptol)**	EO	De Pooter et al., 1987 [61]; Sarer and Konuklugil, 1996 [104]; Chalchat et al., 1998 [60]; Kökdil et al., 1998a [54]; Kobaisy et al., 2005 [8]; Malenčić et al., 2008 [105]; Alim et al., 2009 [56]; Mancini et al., 2009 [99]; Gkinis et al., 2010 [14]; Kilic et al., 2011 [55]; Bozari et al., 2013 [101]; Bozok et al., 2017 [4]; Narimani et al., 2017 [13]; Akbaba et al., 2021 [100]; Aćimović et al., 2022 [62]; Semïz et al., 2022 [106]
	MeOH	Sarikurkcu et al., 2019 [5]
**Dehydro-1,8-cineole**	EA	Zaharieva et al., 2023 [11]
	EO	Kobaisy et al., 2005 [8]
***α*-Pinene**	EO	De Pooter et al., 1987 [61]; Kökdil et al., 1998a [54]; Chalchat et al., 1998 [60]; Kobaisy et al., 2005 [8]; Gkinis et al., 2010 [14]; Kilic et al., 2011 [55]; Semïz et al., 2022 [106]
**Sabinene**	EO	De Pooter et al., 1987 [61]; Chalchat et al., 1998 [60]; Kökdil et al., 1998a [54]; Kobaisy et al., 2005 [8]; Gkinis et al., 2010 [14]; Kilic et al., 2011 [55]; Aćimović et al., 2022 [62]
	EA	Zaharieva et al., 2023 [11]
***β*-Pinene**	EO	De Pooter et al., 1987 [61]; Chalchat et al., 1998 [60]; Kökdil et al., 1998a [54]; Alim et al., 2009 [56]; Kilic et al., 2011 [55]; Aćimović et al., 2022 [62]; Semïz et al., 2022 [106]
	EA	Zaharieva et al., 2023 [11]
**Myrcene**	EO	De Pooter et al., 1987 [61]; Chalchat et al., 1998 [60]; Kökdil et al., 1998a [54]; Alim et al., 2009 [56]; Gkinis et al., 2010 [14]; Aćimović et al., 2022 [62]; Semïz et al., 2022 [106]
	EA	Zaharieva et al., 2023 [11]
**Limonene**	EO	De Pooter et al., 1987 [61]; Kökdil et al., 1998a [54]
	EA	Zaharieva et al., 2023 [11]
**Terpineol**	EO	De Pooter et al., 1987 [61]; Akbaba et al., 2021 [100]; Semïz et al., 2022 [106]
***β*-Ocimene**	EO	Kökdil et al., 1998a [54]; Kobaisy et al., 2005 [8]; Gkinis et al., 2010 [14]; Semïz et al., 2022 [106]
	EA	Zaharieva et al., 2023 [11]
***γ*-Terpinene**	EO	Kökdil et al., 1998a [54]; Kobaisy et al., 2005 [8]; Gkinis et al., 2010 [14]; Aćimović et al., 2022 [62]; Semïz et al., 2022 [106]
***p*-Mentha-2,4(8)-diene**	EO	Kobaisy et al., 2005 [8]
**Pinocarvone**	EO	Kobaisy et al., 2005 [8]
**Terpinen-4-ol**	EO	Kökdil et al., 1998a [54]; Kobaisy et al., 2005 [8]; Semïz et al., 2022 [106]

***α*-Terpineol**	EO	Kökdil et al., 1998a [54]; Kobaisy et al., 2005 [8]
**Myrtenal**	EO	Kobaisy et al., 2005 [8]; Gkinis et al., 2010 [14]; Aćimović et al., 2022 [62]
**Carvone**	EO	Kobaisy et al., 2005 [8]; Kökdil et al., 1998a [54]
**Geraniol**	EO	Kobaisy et al., 2005 [8]
***α*-Thujene**	EO	Gkinis et al., 2010 [14]
***p*-Cymene**	EO	Gkinis et al., 2010 [14]; Semi̇z et al., 2022 [106]
***cis*-Sabinene hydrate**	EO	Gkinis et al., 2010 [14]
**Terpinolene**	EO	Gkinis et al., 2010 [14]
**Linalool**	EO	Kökdil et al., 1998a [54]; Gkinis et al., 2010 [14]; Akbaba et al., 2021 [100]; Semi̇z et al., 2022 [106]
**Pinocarveol**	EO	Kökdil et al., 1998a [54]
**Pulegone**	EO	Handjieva et al., 1996 [57]; Mancini et al., 2009 [99]; Narimani et al., 2017 [13]
**Camphene**	EO	Kilic et al., 2011 [55]; Alim et al., 2009 [56]
**Camphor**	EO	Kilic et al., 2011 [55]; Semi̇z et al., 2022 [106]
**Borneol**	EO	Kilic et al., 2011 [55]; Semi̇z et al., 2022 [106]
**Tricyclene**	EO	Semi̇z et al., 2022 [106]
**3-Carene**	EO	Semi̇z et al., 2022 [106]
	Sesquiterpenes	
***β*-Caryophyllene**	EO	De Pooter et al., 1987 [61]; Handjieva et al., 1996 [57]; Kökdil et al., 1998a [54]; Kobaisy et al., 2005 [8]; Malenčić et al., 2008 [105]; Gkinis et al., 2010 [14]; Bozari et al., 2013 [101]; Akbaba et al., 2021 [100]
**14-Hydroxy-9-*epi*-(E)- caryophyllene**	EO	Gkinis et al., 2010 [14]
**14-Hydroxy-*δ*-cadinene**	EO	Kobaisy et al., 2005 [8]
**14-Oxy-*α*-muurolene**	EO	Kobaisy et al., 2005 [8]
**Allo-aromadendrene**	EO	Kökdil et al., 1998a [54]; Kobaisy et al., 2005 [8]
**Aromadendrene**	EO	Kökdil et al., 1998a [54]; Gkinis et al., 2010 [14]; Bozari et al., 2013 [101]
**Bicyclogermacrene**	EO	Kobaisy et al., 2005 [8]; Bozari et al., 2013 [101]
	EA	Zaharieva et al., 2023 [11]
**Cadinene**	EO	De Pooter et al., 1987 [61]; Kökdil et al., 1998a [54]; Gkinis et al., 2010 [14]; Bozari et al., 2013 [101]; Aćimović et al., 2022 [62]
**Caryophyllene**	EO	Bozari et al., 2013 [101]
	EA	Zaharieva et al., 2023 [11]
**Caryophyllene oxide**	EO	De Pooter et al., 1987 [61]; Sarer and Konuklugil, 1996 [104]; Chalchat et al., 1998 [60]; Gkinis et al., 2010 [14]; Bozok et al., 2017 [4]; Narimani et al., 2017 [13]; Akbaba et al., 2021 [100]; Aćimović et al., 2022 [62]; Semi̇z et al., 2022 [106]
**Cedr-8-(15)-en-9-*α*-ol**	EO	Kobaisy et al., 2005 [8]
**Cedrol**	EO	Kobaisy et al., 2005 [8]

***cis*-Calamenen-10-ol**	EO	Gkinis et al., 2010 [14]
***cis*-murola-4(14),5-diene**	EO	Kobaisy et al., 2005 [8]
**Copaene**	EO	De Pooter et al., 1987 [61]; Kökdil et al., 1998a [54]; Bozari et al., 2013 [101]
**Cubenol**	EO	Bozari et al., 2013 [101]
**Elemol**	EO	Kökdil et al., 1998a [54]; Gkinis et al., 2010 [14]; Bozari et al., 2013 [101]; Gormez et al., 2013 [6]; Narimani et al., 2017 [13]; Akbaba et al., 2021 [100]
**Epi-*α*-muurolol**	EO	Kobaisy et al., 2005 [8]
**Farnesol**	EO	Kökdil et al., 1998a [54]; Semïz et al., 2022 [106]
**Geijerene**	MeOH	Sarikurkcu et al., 2019 [5]
**Germacrene**	EO	Bozari et al., 2013 [101]
**Germacrene-D**	EO	De Pooter et al., 1987 [61]; Chalchat et al., 1998 [60]; Kökdil et al., 1998a [54]; Kobaisy et al., 2005 [8]; Malenčić et al., 2008 [105]; Alim et al., 2009 [56]; Gkinis et al., 2010 [14]; Kilic et al., 2011 [55]; Bozok et al., 2017 [4]; Narimani et al., 2017 [13]; Akbaba et al., 2021 [100]; Aćimović et al., 2022 [62]; Semïz et al., 2022 [106]
	EA	Zaharieva et al., 2023 [11]
**Globulol**	EO	Kökdil et al., 1998a [54]
**Hinesol acetate**	EO	Kobaisy et al., 2005 [8]
**Humulene**	EA	Zaharieva et al., 2023 [11]
**Humulene epoxide II**	EO	Kobaisy et al., 2005 [8]; Gkinis et al., 2010 [14]
**Humulene oxide**	EO	Kökdil et al., 1998a [54]
**Iso-longifolol**	EO	Kobaisy et al., 2005 [8]
**Khusinol**	EO	Kobaisy et al., 2005 [8]
**Ledol**	EO	Kökdil et al., 1998a [54]
**Longifolene**	EO	Kobaisy et al., 2005 [8]
**Nerolidol**	EO	Sarer and Konuklugil, 1996 [104]
**Oplopanone**	EO	Kobaisy et al., 2005 [8]
**Palustrol**	EO	Akbaba et al., 2021 [100]
**Sesquiphellandrene**	EO	Handjieva et al., 1996; Gkinis et al., 2010
**Spathulenol**	EO	Sarer and Konuklugil, 1996 [104]; Chalchat et al., 1998 [60]; Kökdil et al., 1998a [54]; Kobaisy et al., 2005 [8]; Malenčić et al., 2008 [105]; Mancini et al., 2009 [99]; Bozari et al., 2013 [101]; Gormez et al., 2013 [6]; Narimani et al., 2017 [13]; Akbaba et al., 2021 [100]
**T-Cadinol**	EO	Kökdil et al., 1998a [54]
***trans*-Caryophyllene**	EO	Sarer and Konuklugil, 1996 [104]; Chalchat et al., 1998 [60]; Alim et al., 2009 [56]; Kilic et al., 2011 [55]; Gormez et al., 2013 [6]; Bozok et al., 2017 [4]; Narimani et al., 2017 [13]; Aćimović et al.,

		2022 [62]
**Valerianol**	EO	Kobaisy et al., 2005 [8]
**Viridiflorol**	EO	Kökdil et al., 1998a [54]
***α*-Cadinol**	EO	Kökdil et al., 1998a [54]; Akbaba et al., 2021 [100]
***α*-Calacorene**	EO	Gkinis et al., 2010 [14]
***α*-Copaene-11-ol**	EO	Kökdil et al., 1998a [54]
***α*-Copaene-8-ol**	EO	Kökdil et al., 1998a [54]
***α*-Gurjunene**	EA	Zaharieva et al., 2023 [11]
***α*-Humulene**	EO	Chalchat et al., 1998 [60]; Kökdil et al., 1998a [54]; Alim et al., 2009 [56]; Mancini et al., 2009 [99]; Gkinis et al., 2010 [14]; Bozari et al., 2013 [101]; Narimani et al., 2017 [13]; Semïz et al., 2022 [106]
***α*-Muurolene**	EO	Kobaisy et al., 2005 [8]
***α*-neo-Clovene**	EO	Kobaisy et al., 2005 [8]
***β*-Acoradienol**	EO	Kobaisy et al., 2005 [8]
***β*-Bisabolene**	EO	Handjieva et al., 1996 [57]; Chalchat et al., 1998 [60]; Kobaisy et al., 2005 [8]; Mancini et al., 2009 [99]; Gkinis et al., 2010 [14]; Bozok et al., 2017 [4]; Akbaba et al., 2021 [100]
***β*-Bourbonene**	EO	De Pooter et al., 1987 [61]; Gkinis et al., 2010 [14]; Bozari et al., 2013 [101]; Aćimović et al., 2022 [62]; Semïz et al., 2022 [106]
	EA	Zaharieva et al., 2023 [11]
***β*-Caryophyllene oxide**	EO	Kökdil et al., 1998a [54]; Kobaisy et al., 2005 [8]; Handjieva et al., 1996 [57]
***β*-Copaene**	EA	Zaharieva et al., 2023 [11]
***β*-Elemene**	EO	De Pooter et al., 1987 [61]; Kökdil et al., 1998a [54]; Bozari et al., 2013 [101]; Aćimović et al., 2022 [62]; Semïz et al., 2022 [106]
	EA	Zaharieva et al., 2023 [11]
***β*-Eudesmol**	EO	Kökdil et al., 1998a [54]
***β*-Farnesene**	EO	De Pooter et al., 1987 [61]; Handjieva et al., 1996 [57]; Kökdil et al., 1998a [54]; Gkinis et al., 2010 [14]; Semïz et al., 2022 [106]
***β*-Gurjunene**	EO	Kobaisy et al., 2005 [8]; Gkinis et al., 2010 [14]; Bozari et al., 2013 [101]
***β*-Sesquiphellandrene**	EO	Kökdil et al., 1998a [54]
***γ*-Muurolene**	EO	Kobaisy et al., 2005 [8]

EA – ethyl acetate; EO - essential oil; HE – hexane extract; AE acetone extract; MeOH – methanolic extract, EtOH – ethanol extract; WE – water extract.

Table S2. Peak areas of compounds identified using QToF MS in eleven different *Nepeta nuda* leaf extracts.

No	Compound name	Selište	Brodica	Debeli Lug	Rtanj	Straža	Vinatovača	Vlasina	Donji Krivodol	Janjska reka	Židilje	Gornje Selo
						Hydroxybenzoic acid derivatives						
**1**	**Galloyl hexoside**	68827419	49816429	79828266	73984855	78642392	67790250	1025496	96043299	151584704	9944625	116560317
**2**	**Gallic acid**	NF	NF	NF	666419	641442	NF	NF	861967	NF	NF	347965
**3**	**Dihydroxybenzoic acid**	NF	NF	NF	NF	NF	NF	NF	2981235	1582865	NF	NF
**4**	**Dihydroxybenzoic acid hexoside**	63411934	49721319	25869910	66379401	49791202	38673114	35114408	73940725	95890976	83266823	50583519
**5**	**Vanillic acid hexoside**	5096132	9828399	3759343	4750432	8596501	2703691	6793888	15344418	14002208	16205044	9491045
**6**	**Hydroxybenzoic acid**	24508576	79935361	7886827	14440272	19071606	8408490	56529193	37886718	14715451	102528578	7924664
**7**	**Dihydroxybenzoic acid hexoside isomer**	12954831	NF	NF	12132645	6527028	6661118	NF	4129682	4820412	25901016	NF
**8**	**Dihydroxybenzoic acid isomer**	19658394	4064622	12551063	13480677	17322582	7576350	841385	10869299	6196600	14342630	4484404
**9**	**Vanillic acid**	NF	NF	NF	NF	NF	NF	NF	NF	NF	27164940	NF
**10**	**Hydroxybenzoic acid hexoside**	33194428	14106799	21334299	33791934	17046752	29033835	14280943	10419373	9936991	45087692	16336170
						Hydroxycinnamic acid derivatives						
**11**	**Syringic acid hexoside**	29706413	33543218	8696094	5630572	12505794	11710762	NF	40657590	13074155	11966385	8047841
**12**	**Caffeoyltartaric acid**	40785804	NF	NF	16568466	3612252	14081695	3554261	15876633	10361020	10008515	8970271
**13**	**Caffeoyltartronic acid**	53416241	209866014	39981858	43287883	44433410	70356425	35580171	19515495	152252095	37020720	80246154
**14**	**Caffeic acid hexoside**	NF	NF	NF	NF	NF	NF	5539026	16505840	8484834	6743288	7775625
**15**	**Caffeoylglycolic acid**	58168302	141123128	31016355	85825889	91553739	87743135	28306582	33705890	114860323	44433768	121946874
**16**	**Umbelliferone**	59483038	80804701	59597102	55754706	59831867	86395479	48349032	39080170	141986798	53406341	110779397
**17**	**Caffeoyltartronic acid isomer**	161398227	184313820	126013508	166047694	160487978	191467841	119735953	117874185	245135613	151786278	182440956
**18**	**Sinapic acid**	5140121	1035830	6546941	11896636	2309679	10746993	NF	NF	3996981	NF	1338253
**19**	**Caffeic acid hexoside isomer**	NF	9274609	6363094	7692410	9580335	7946026	1878689	11355444	10668173	4505813	11061228
**20**	**Dihydrocaffeic acid hexoside**	3754961	5503630	4004414	4348926	6040352	3410555	1821104	5120358	3581450	3146137	2646418
**21**	**Caffeic acid**	22634446	24737043	16377622	16146058	20981301	17556206	NF	34342711	20628972	NF	6202734
**22**	**Feruloyltartaric acid**	33093009	NF	NF	24356075	NF	NF	3583088	47545873	5842950	3431502	NF
**23**	**Ethyl caffeate**	114910260	64749527	60921967	100382768	61146940	63604577	77670595	88896805	98859092	54107105	110553041

**24**	**Caffeoylmalic acid**	210267762	164098824	111186310	380481585	151365388	144450892	152314740	211795201	199124003	148005607	161803742
**25**	**Feruloylmalic acid**	27143201	15653101	21628843	21992137	9499374	12330849	9446397	49854724	30176239	10983446	10567449
**26**	**Rosmarinic acid**	97839486	107208254	82019387	79263330	107171359	97070097	NF	89021322	110638581	62039338	104245548
**27**	**Ferulic acid**	13367694	10490861	8399330	11715530	14443554	18751777	7515372	9589311	18913418	13435876	10183613
**28**	**Clinopodic acid A**	NF	NF	NF	NF	NF	NF	NF	NF	NF	NF	16306924
**29**	**Nepetoidin A or B**	NF	NF	NF	NF	10052962	NF	NF	8961568	33059163	34868387	NF
						Iridoid glycosides						
**30**	**Aucubin**	3019416	2689171	5338978	3019459	3255214	4637743	3064093	4435849	6832249	3845968	3279941
**31**	**6α-Hydroxyadoxoside**	19484203	13488419	6568908	14283131	36467093	6616257	31729833	13479475	11468254	5977208	40606265
**32**	**Nepetanudoside C + HCOOH**	5537833	11181290	29067501	15046558	19400049	8908500	8230991	7942952	15183005	18881823	5862898
**33**	**5-Deoxylamiol**	19593137	24494721	24957926	9936655	50436747	40817816	20464364	NF	20122951	23185789	15044820
**34**	**1,5,9-*epi*-Deoxyloganic acid hexoside + HCOOH**	9912956	7771022	6748864	2582677	24392436	10354180	6756417	10481429	18632018	14873179	5864643
**35**	**Nepetanudoside A + HCOOH**	219831446	204795379	173077488	115271002	NF	146761959	209497923	205541731	205357373	188145894	275430921
**36**	**1,5,9-*epi*-Deoxyloganic acid**	111955667	76169497	73617210	79031859	97131270	154857663	90398682	20053831	62831952	90858335	49198745
**37**	**Nepetaside**	3395628	16623346	3363498	4191534	2848206	3619512	14428017	NF	3689419	7953326	6265567
**38**	**6-Deoxylamioside**	5048880	5256522	3966211	2575392	3486690	4906205	6195053	6237366	7057259	5113908	5518474
						Iridoid aglycones						
**39**	**Genipin**	8865495	NF	NF	NF	NF	NF	NF	6460335	NF	NF	12899143
**40**	**5,9-Dehydronepetalactone**	8058755	NF	7486301	3472251	21689929	17255035	7265040	4539049	7807331	11149421	4756406
**41**	**Nepetaside aglycone acetate**	54099027	70435147	52541529	62745360	63223568	63927228	59681311	NF	46338721	73924859	75690546
**42**	**Loganetin**	88517703	91295588	81247238	98334527	NF	65664042	107839654	100917123	100705964	84788881	102136704
**43**	**Deoxygeniposide aglycone**	77828214	86499150	67312571	52223239	NF	60143136	78194774	114290918	126285108	13720293	85576134
**44**	**Nepetalactol**	23303592	16502440	NF	13825490	18481582	NF	18522741	17091497	22562544	17815600	NF
**45**	**7-Deoxyloganetic acid**	121019548	75925978	96813941	72097502	118181110	127088860	102196575	10291008	53206251	93304101	38923416
**46**	**Nepetaracemoside B aglycone**	64747741	50298886	67645008	43946505	64888731	82791929	59128004	NF	27033511	47448657	14968975
**47**	**Nepetalactone**	5081237	NF	NF	4366095	NF	2247981	6294609	NF	NF	NF	NF
**48**	**De-4-methylnepetalactol**	3092473	1868459	1448789	2858439	NF	NF	3749565	2492500	2404847	NF	2805553

**49**	**7-Deoxyloganetin**	NF	864478	NF	NF	NF	NF	NF	NF	NF	NF	NF
**50**	**Nepetalic acid**	2105121	963989	583051	990798	922668	2013698	1930162	631680	1514519	1196966	736319
**51**	**Dihydronepetalactone**	NF	5142957	NF	NF	NF	NF	NF	NF	2283601	NF	NF
**52**	**Nepetalactone**	NF	NF	NF	1456878	NF	NF	1899632	NF	1877026	NF	940246
**53**	**Nepetalactol acetate**	5085035	9415261	4807244	NF	5041691	NF	NF	8031114	NF	NF	5300142
**54**	**Nepetalactone**	5706798	NF	NF	NF	NF	26674714	NF	NF	NF	NF	2164527
Flavonoid glycosides												
**55**	**Luteolin 7-*O*-(6"-hexosyl)-hexuronide**	NF	5728957	NF	2777467	5683670	3079868	4487511	12142195	10280978	5619287	10176381
**56**	**Luteolin 7-*O*-(2"-hexuronyl)-hexuronide**	8378788	NF	8584864	15804190	NF	12241555	NF	12683796	NF	NF	NF
Flavonoid aglycones												
**57**	**Thymusin**	NF	NF	7723680	15217674	NF	7261445	NF	12273791	NF	12590588	3187246
**58**	**Chrysoeriol**	NF	NF	NF	NF	NF	11940081	NF	6419231	NF	NF	NF
**59**	**Cirsimaritin**	32215074	NF	41417833	28527724	36441038	58175168	16470535	74323702	10551524	59829445	11404089
**60**	**Xanthomicrol**	27315930	11283766	6945961	45626248	22559361	19578337	3689246	9379672	6387221	17857379	10261790
**61**	**Acacetin**	NF	NF	NF	NF	NF	21219670	NF	13980521	NF	13393970	NF
**62**	**Salvigenin**	259848289	113593466	140176896	149531432	180503029	239060447	140641534	133228890	122340436	199990146	137798708
Other metabolites												
**63**	**Quinic acid**	12638782	4345002	13867590	NF	2850656	10717683	80343463	6945857	5026079	31408322	1630708
**64**	**3-(3,4-Dihydroxyphenyl)-lactic acid**	18023840	11383440	14114567	6543041	11255499	13742909	NF	33485089	39092982	3953959	24653454
**65**	**Hexenyl-pentosyl-hexoside**	2796589	NF	NF	10831451	51545148	16230725	3855288	NF	NF	11880396	NF
**66**	**Schizonepetin**	8357111	7781772	6585958	NF	18590582	13967535	NF	3827262	6279403	9249708	NF
**67**	**12-*O*-Hexosyl-jasmonate**	78592256	79622350	68539143	81204375	91302772	92381522	88577591	49740313	62038918	75626213	80306694
**68**	**Schizonepetin isomer**	3875462	11557505	NF	4558663	3929896	4118665	4519467	NF	NF	NF	NF
**69**	**2,5-Dimethyl-hexanedioic acid**	18571094	10480083	17055171	18451911	16860753	11191572	16007140	15367235	15494307	11849984	21912657
**70**	**2-Carboxy-3-methyl-cyclopentaneacetic acid**	NF	1173303	NF	890418	1392178	2307865	NF	978045	1084270	828665	1011595
**71**	**Argolic acid A rhamnoside**	19652507	9266785	14805373	16841551	13887886	16895772	19891487	8523679	9101520	11879339	14577691
**72**	**2-Carboxy-α,3-dimethyl-cyclopentaneacetic acid**	4100956	1446551	3165395	3718776	2754972	2575205	3048148	1869599	1989732	1722892	2199813

**73**	**9-Oxononanoic acid**	NF	48791557	61090022	60535187	36035201	77607432	67750156	71017852	62100259	44586670	77357382
**74**	**2-Carboxy-3-methyl-cyclopentaneacetic acid isomer**	NF	NF	8705348	9371719	12861977	9242889	9384400	6824961	10857976	10881622	9528147
**75**	**Argolic acid A**	14895035	11225966	12679788	14442303	15948198	14134338	10740518	8651484	9957215	NF	14439798
**76**	**Argolic acid A methyl ether rhamnoside**	49438884	23945371	10207828	15343403	13304173	14361581	10788595	5777299	10869489	14588601	12447571
**77**	**Nepetonic acid**	47887031	46455500	49155212	45408988	29125191	58594330	51375418	53182891	47908818	34023140	59445119
**78**	**2-Carboxy-α,3-dimethyl-cyclopentaneacetic acid dimethyl ester**	328977	6316302	NF	NF	NF	NF	NF	NF	NF	NF	4917518

NF – not found.

Table S3. Validation parameters of the SRM method of the UHPLC/DAD/(±)HESI-MS^2 for the quantification of targeted compounds.

t_R, min	Compounds	Ionisation mode	Molecular ion, *m/z*	MS/MS fragments, *m/z* (Collision energy, *eV*)	R^2	LOD, mg/L	LOQ, mg/L	Regression equation, Y = A + B*X	DAD spectra λmax (nm)
1.64	**Chlorogenic acid**	**$[M - H]^-$**	353	191 (25)	0.9999	0.01	0.04	Y = 48951 + 721*X	230, 330
2.65	**Caffeic acid**	**$[M - H]^-$**	179	135 (18); 107 (18)	0.9972	0.53	1.78	Y = - 199.31 + 265.22*X	230, 330
3.43	**Loganin**	**$[M + HCOOH - H]^-$**	435	227 (20), 124 (20)	0.9999	0.13	0.45	Y = 5785 + 101*X	220
4.22	**Rutin**	**$[M - H]^-$**	609	301 (32); 271 (58)	0.9994	0.14	0.48	Y = 2700 + 167*X	270, 300, 340
4.26	**Rosmarinic acid**	**$[M - H]^-$**	359	161 (30), 133 (30)	0.9937	0.09	0.31	Y = 4778 + 1322470*X	240, 330
5.12	**Astragalin**	**$[M - H]^-$**	447	284 (29); 255 (43)	0.9976	0.30	1.00	Y = 26211 + 476*X	270, 360
5.52	**1,5,9-*epi*-Deoxyloganic acid**	**$[M + HCOOH - H]^-$**	405	359 (20), 197 (20)	0.9983	1.00	3.32	Y = 106225 + 967*X	240
5.70	**Luteolin**	**$[M - H]^-$**	285	175 (27); 134 (36)	0.9956	0.65	2.16	Y = 698354 + 4565410*X	260, 360
6.07	**Apigenin**	**$[M - H]^-$**	269	225 (23); 151 (26)	0.9911	0.65	2.16	Y = 35609 + 1488540*X	270, 360
6.20	**Isorhamnetin**	**$[M - H]^-$**	315	300 (30); 151 (25)	0.9978	0.07	0.25	Y = - 7297 + 300*X	280, 330
6.35	**5,9-Dehydronepetalactone**	**$[M + H]^+$**	165	149 (10), 77 (10)	0.9967	1.42	4.75	Y = - 1009.05 + 1.26*X	300
7.60	***cis,trans*-Nepetalactone**	**$[M + H]^+$**	167	111 (10), 77 (10)	0.9948	0.54	1.81	Y = - 4261.36 + 17.13*X	220

LOD – limit of detection; LOQ – limit of quantification; t_R – retention time.

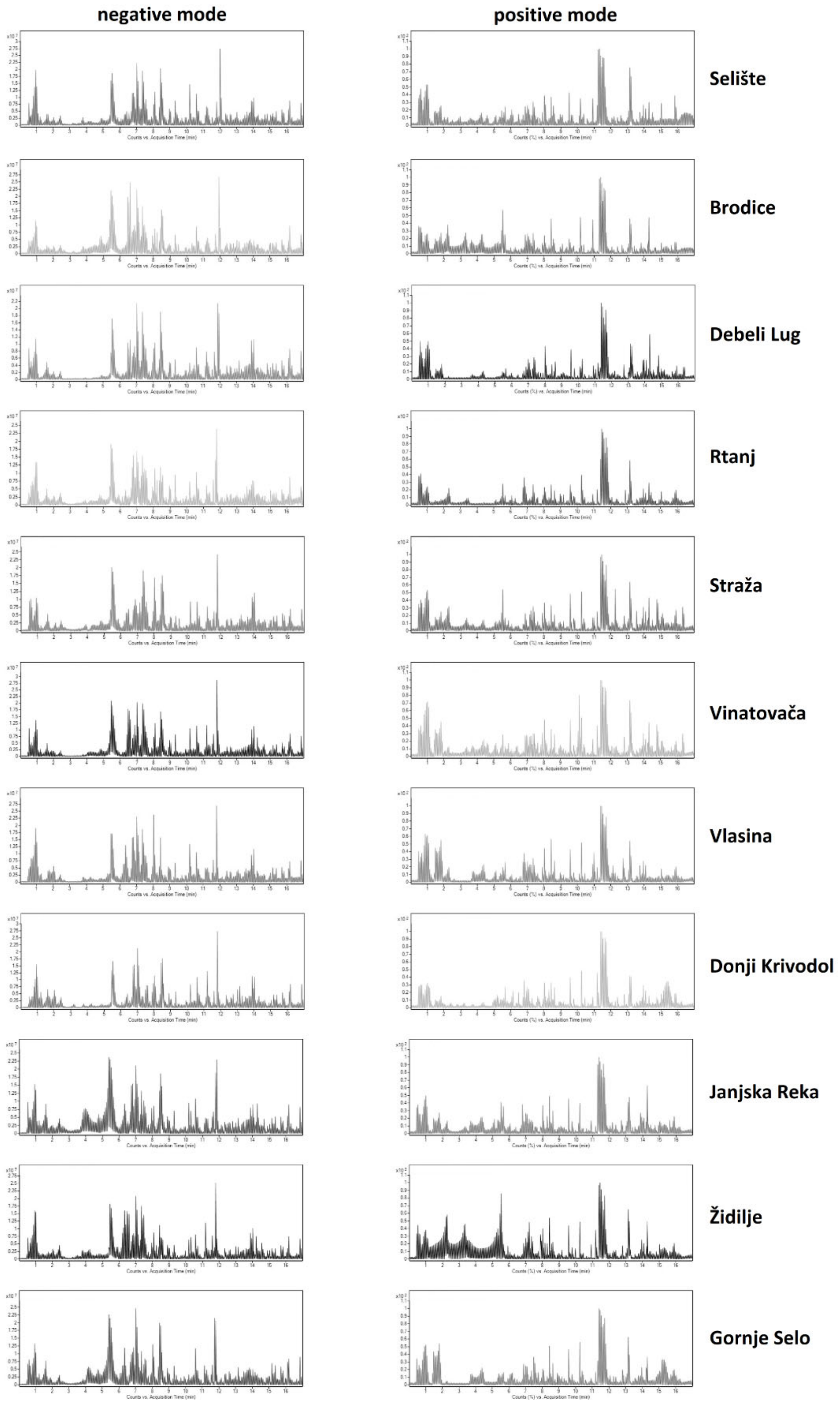

Figure S1. Base peak chromatograms, in negative and positive ionization modes, of eleven different *Nepeta nuda* leaf extracts; for peak annotation see retention times in Table 1 and peak areas from Table S2.

A

B

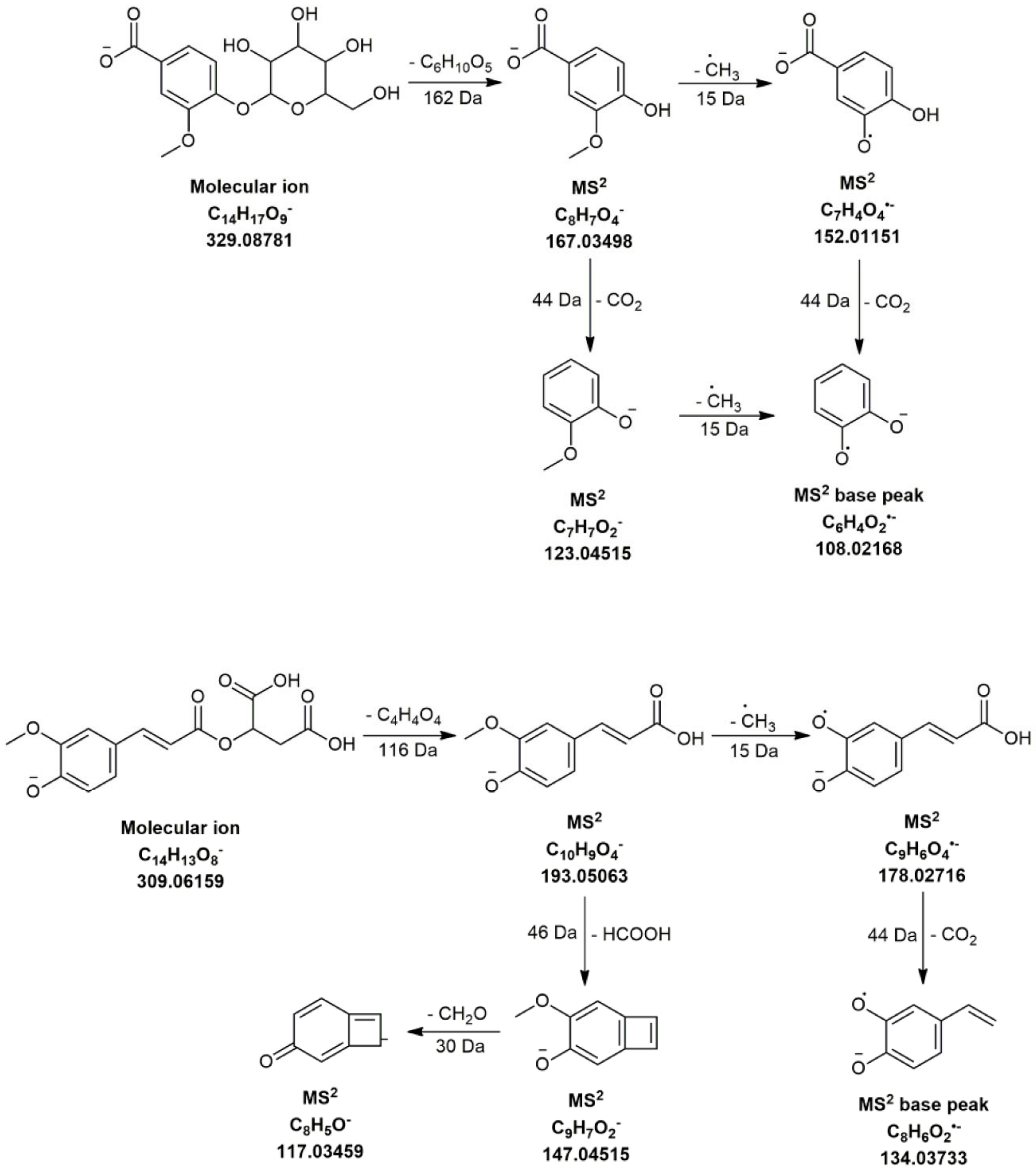

Figure S2. A proposed fragmentation pathway of **(A)** compound **5** (vanillic acid hexoside) and **(B)** compound **25** (feruloylmalic acid).

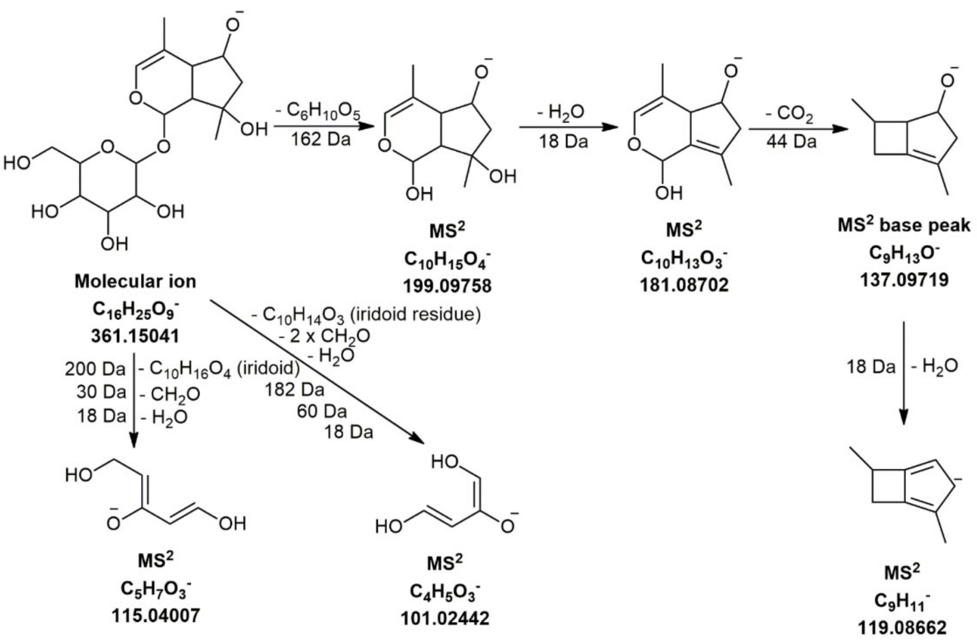

Figure S3. A proposed fragmentation pathway of compound **33** (5-deoxylamiol).

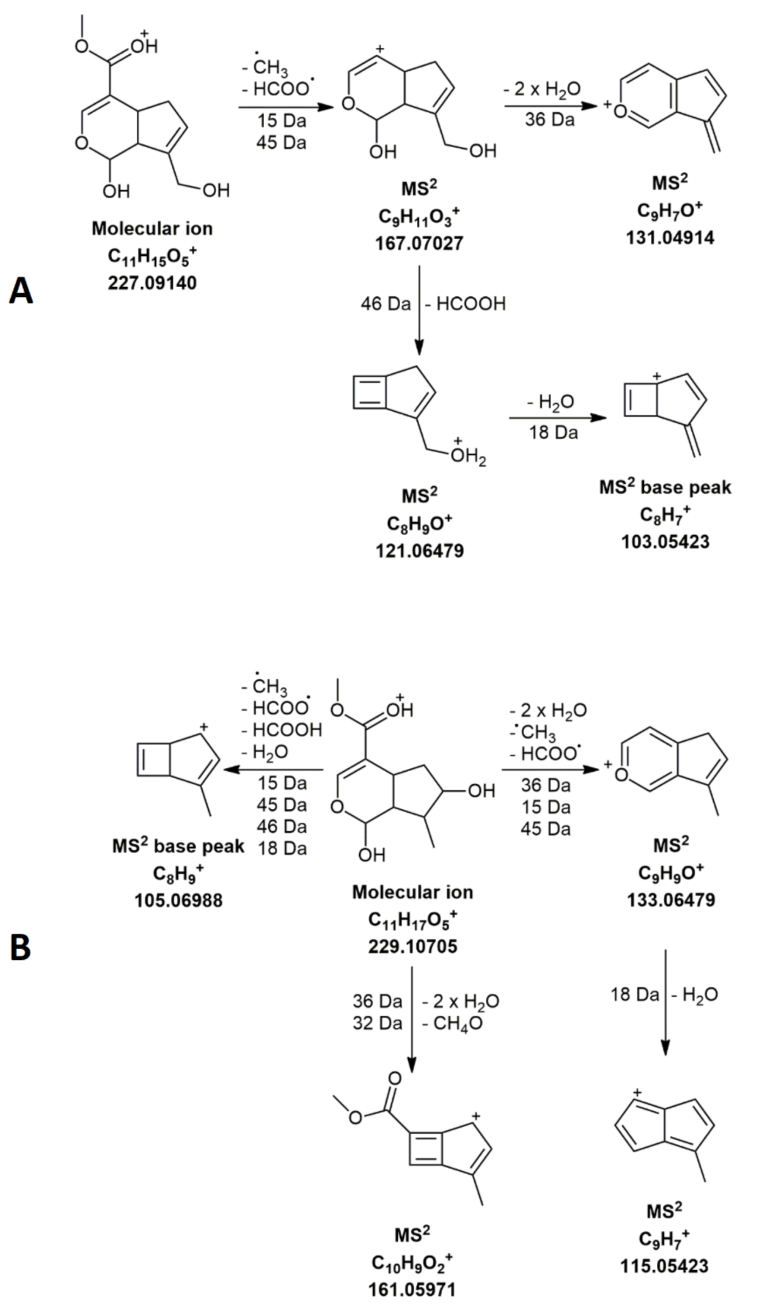

Figure S4. A proposed fragmentation pathway of **(A)** compound **39** (genipin) and **(B)** compound **42** (loganetin).

A

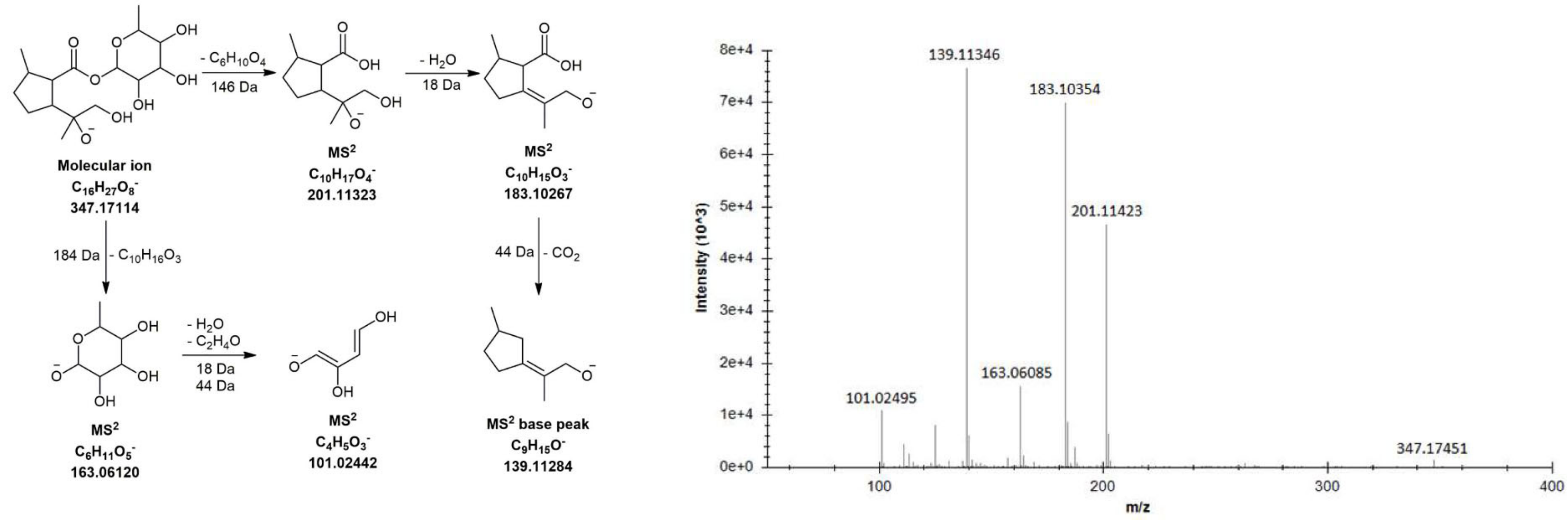

B

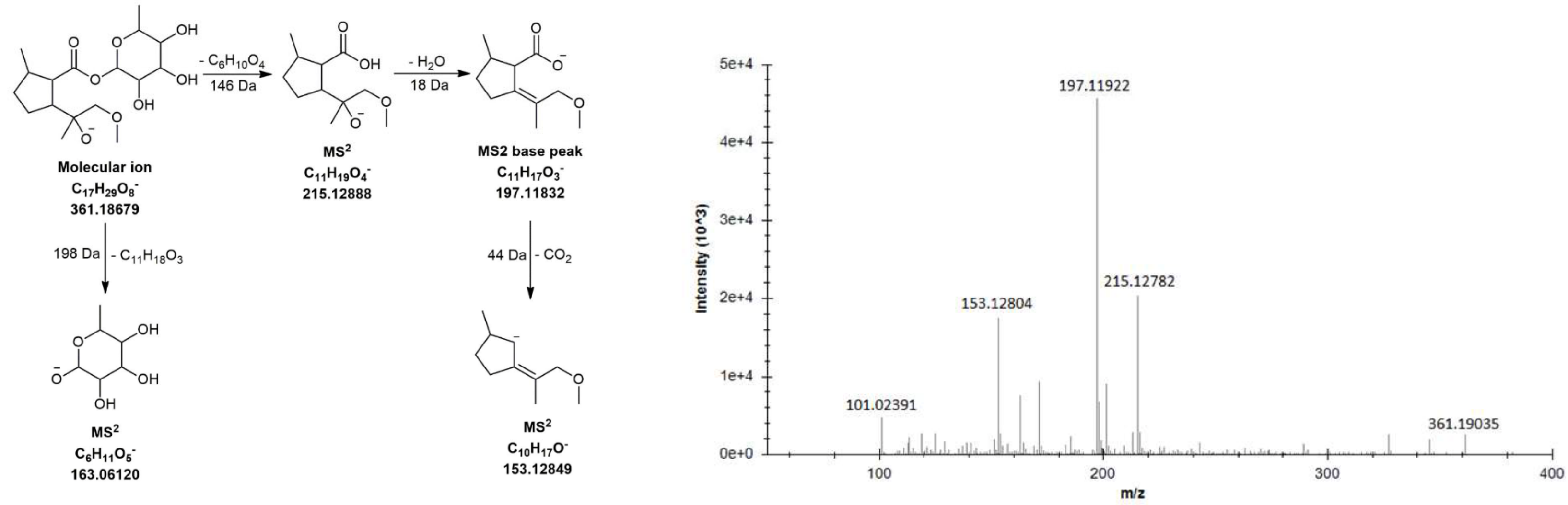

Figure S5. A proposed fragmentation pathway and MS² spectra of **(A)** compound **71** (argolic acid A rhamnoside) and **(B)** compound **76** (argolic acid A methyl ether rhamnoside).
